# Oxygen induced promotion of electrochemical reduction of CO_2_ via co-electrolysis

**DOI:** 10.1038/s41467-020-17690-8

**Published:** 2020-07-31

**Authors:** Ming He, Chunsong Li, Haochen Zhang, Xiaoxia Chang, Jingguang G. Chen, William A. Goddard, Mu-jeng Cheng, Bingjun Xu, Qi Lu

**Affiliations:** 1 State Key Laboratory of Chemical Engineering, Department of Chemical Engineering, Tsinghua University, Beijing, 100084 China; 2 Center for Catalytic Science and Technology, Department of Chemical and Biomolecular Engineering, University of Delaware, Newark, DE 19716 USA; 3 Department of Chemical Engineering, Columbia University, New York, NY 10027 USA; 4 Materials and Process Simulation Center, California Institute of Technology, Pasadena, CA 91125 USA; 5 Department of Chemistry, National Cheng Kung University, 701 Tainan, Taiwan

**Keywords:** Catalytic mechanisms, Electrocatalysis, Electrocatalysis

## Abstract

Harnessing renewable electricity to drive the electrochemical reduction of CO_2_ is being intensely studied for sustainable fuel production and as a means for energy storage. Copper is the only monometallic electrocatalyst capable of converting CO_2_ to value-added products, e.g., hydrocarbons and oxygenates, but suffers from poor selectivity and mediocre activity. Multiple oxidative treatments have shown improvements in the performance of copper catalysts. However, the fundamental underpinning for such enhancement remains controversial. Here, we combine reactivity, in-situ surface-enhanced Raman spectroscopy, and computational investigations to demonstrate that the presence of surface hydroxyl species by co-electrolysis of CO_2_ with low concentrations of O_2_ can dramatically enhance the activity of copper catalyzed CO_2_ electroreduction. Our results indicate that co-electrolysis of CO_2_ with an oxidant is a promising strategy to introduce catalytically active species in electrocatalysis.

## Abstract (web summary)

While the electrochemical conversion of CO_2_ to highly reduced products is unique to copper, there are still gaps in understanding copper catalysts’ efficacy. Here, authors find that co-electrolysis of CO_2_ with O_2_ can enhance copper’s catalytic activities.

## Introduction

Among all electrocatalysts explored to date, copper (Cu) exhibits the unique capability in reducing CO_2_ to valuable hydrocarbons and oxygenates. However, significant enhancement in the rate and selectivity for valuable products and in energy efficiency for Cu-based electrocatalysis remains imperative for this strategy to become industrially viable^1–3^. Oxidative treatments of polycrystalline Cu surfaces have been shown to improve the activity and selectivity towards the production of value-added hydrocarbons and oxygenates, for example, thermal oxidation followed by reduction (so called oxide-derived Cu)^4,5^, oxygen plasma activation^6^, and anodic oxidation^7–9^. Although there is a general recognition of the beneficial effect of oxidative treatments on Cu-based catalysts in the CO_2_ reduction reaction (CO_2_RR), the mechanisms through which the enhancement is realized remain a topic of considerable discussion. A key point of debate is whether oxygen-containing species, e.g., CuO_*x*_, CuO_*x*_(OH)_*y*_, and Cu(OH)_*x*_, are present at the CO_2_RR conditions^6,7,10–19^. Multiple ex situ and in situ/operando characterizations lead to contradicting conclusions^6,7,10–17,19^. As ex situ measurements have the potential of exposing the sample to the ambient condition, the origin of the oxygen-containing species on the Cu surface detected by these methods is uncertain. Another challenge in resolving this debate is the interfacial sensitivity. As the Pourbaix diagram of Cu shows that only metallic Cu should be present at the reducing environment of the CO_2_RR^20^, the Cu oxide and/or hydroxide species are expected to be present only at the electrode/electrolyte interface, if at all. Thus, the detection of these oxidized Cu species hinges upon the interfacial sensitivity of characterization methods. In this regard, recent in situ/operando surface-enhanced Raman spectroscopic investigations provided initial evidence of the existence of oxidized Cu species at reducing potentials^21^. A much less discussed, but arguably more important, aspect is whether the oxidized Cu species, if they indeed exist at the CO_2_RR conditions, contribute to enhanced reactivity of Cu-based catalysts after oxidative treatments. It is conceivable that Cu oxide and/or hydroxide species are mere spectators during the CO_2_RR, while preferentially exposed facets or defects, e.g., located at the grain boundaries on the metallic Cu surface, induced by the treatment are the real cause of change in the catalytic performance^22–25^. This has been shown in our recent work in the CO-reduction reaction on Cu^26^. Thus, establishing a direct correlation between the surface speciation of Cu at reaction conditions and reactivity is a frontier in the CO_2_RR research.

In this work, we demonstrate that the production rate of oxygenates and hydrocarbons in the CO_2_RR is enhanced by up to 216-fold when coupled with the oxygen reduction reaction (ORR) by co-feeding CO_2_ and O_2_ (up to 20%). In situ surface-enhanced Raman spectroscopy (SERS) shows that surface hydroxide species on micron-sized Cu particles are present at the CO_2_RR condition, which are likely formed by ORR. The correlation between the surface hydroxyl species and the enhanced reactivity is supported by additional experimental and computational evidence. The addition of low concentrations of H_2_O_2_, a known possible product of the ORR, in the electrolyte results in much less enhancement of catalytic performance as compared to the case of co-reduction of CO_2_ and O_2_. Importantly, the Raman feature of the surface hydroxyl group is absent, indicating that the presence of the surface hydroxyl group, rather than any oxidant such as H_2_O_2_, is central to the enhanced production rates. In addition, density functional theory (DFT) calculations show the beneficial role of the surface hydroxyl group in reducing the energy barriers in the formation of oxygenates and hydrocarbons. Results reported in this work demonstrate the promise of enhancing the CO_2_RR performance by leveraging coupled reactions via co-electrolysis. From a practical perspective, the strategy of coupling the CO_2_RR and the ORR could reduce the separation cost of trace amounts of O_2_ present in the CO_2_ stream from the flue gas or direct air capture.

## Results

### Co-reduction of CO_2_ with O_2_

**Fig. 1:**
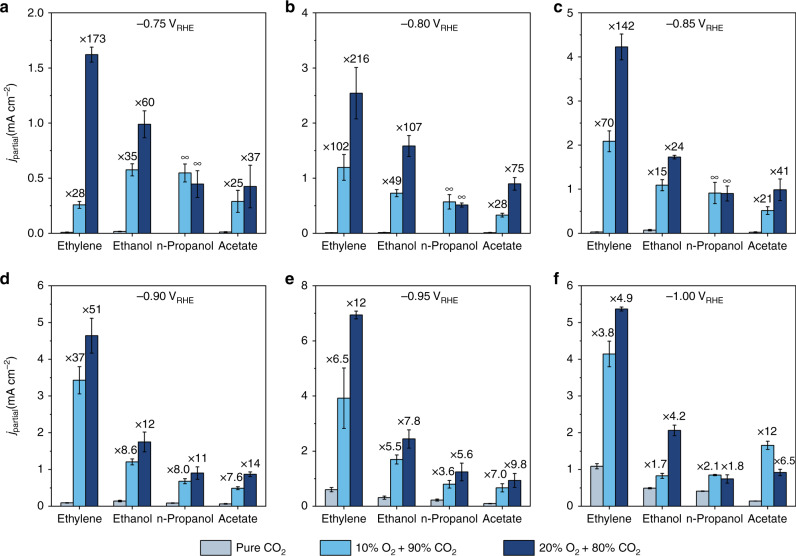
Comparison of C_2+_ product formations. The partial current densities of C_2+_ products measured at 100% CO_2_, 10% O_2_ + 90% CO_2_, and 20% O_2_ + 80% CO_2_ are compared at different potentials of **a** −0.75 V_RHE_, **b** −0.80 V_RHE_, **c** −0.85 V_RHE_, **d** −0.90 V_RHE_, **e** −0.95 V_RHE_, and **f** −1.0 V_RHE_. The numbers stand for the enhancement relative to the rates at pure CO_2_. The error bars represent the standard deviation from at least three independent measurements.

**Fig. 2:**
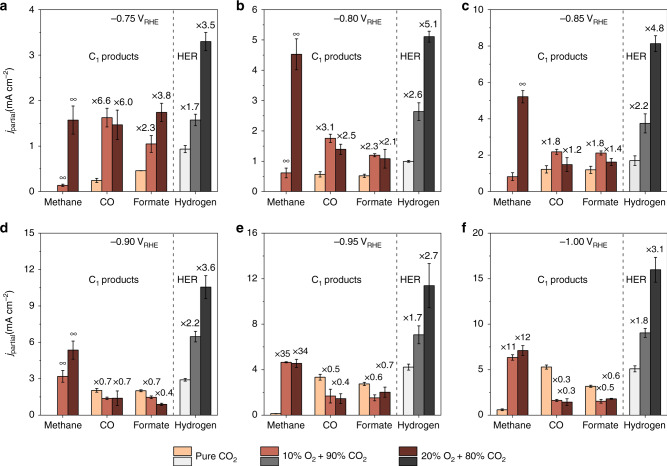
Comparison of C_1_ product and H_2_ formations. The partial current densities of C_1_ products and H_2_ measured at 100% CO_2_, 10% O_2_ + 90% CO_2_, and 20% O_2_ + 80% CO_2_ are compared at different potentials of **a** −0.75 V_RHE_, **b** −0.80 V_RHE_, **c** −0.85 V_RHE_, **d** −0.90 V_RHE_, **e** −0.95 V_RHE_, and **f** −1.0 V_RHE_. The numbers stand for the enhancement relative to the rates at pure CO_2_. The error bars represent the standard deviation from at least three independent measurements.

The electrolysis experiments are conducted in an H-type electrochemical cell with a standard three-electrode setup (Supplementary Fig. 1). Cu electrodes are prepared by depositing commercial polycrystalline Cu powders (~1 μm) (Supplementary Fig. 2) onto a PTFE-treated carbon fiber paper. Compared to planar polycrystalline Cu foil electrodes, these electrodes can effectively improve the mass transport of dissolved gas molecules in H-cell configuration^27–29^. To establish the baseline for the co-reduction studies, the CO_2_RR with pure CO_2_ is conducted in 0.1 M KHCO_3_ (Supplementary Fig. 3a), and the results are consistent with the previous reports on polycrystalline Cu catalysts^29,30^. The major C_2+_ products are ethylene, ethanol, *n*-propanol, and acetate, and the major C_1_ products are methane, CO, and formate. The co-electrolysis is performed by feeding a mixture of CO_2_ and O_2_ with mole ratios of 9:1 and 8:2. The corresponding partial current densities (*j*_partial_) of C_2+_ products, C_1_ products and H_2_ are color-coded in Figs. 1 and 2.

Remarkably, all C_2+_ product formation rates are significantly enhanced in the presence of O_2_ in the gas stream (Fig. 1). At −0.75 V_RHE_ (Fig. 1a), the production rate of ethylene, ethanol and acetate shows a more than 20-fold increase with 10% O_2_ in the feed. With 20% O_2_, the enhancement for ethylene, ethanol and acetate exceeds 170-fold, 55-fold, and 35-fold, respectively. The formation of *n*-propanol is also observed at −0.75 V_RHE_ with a partial current density of ~0.5 mA cm^−^^2^ in the presence of O_2_, while no detectable amount of *n*-propanol is observed at this potential in the oxygen-free atmosphere. The onset potential of *n*-propanol in the oxygen-free atmosphere occurs at as negative as −0.90 V_RHE_ (Fig. 1d), and to achieve a similar rate of ~0.5 mA cm^−^^2^ requires an additional 250 mV overpotential, i.e., −1.00 V_RHE_ (Fig. 1f). Unlike C_2_ products, the formation rate of *n*-propanol does not show a drastic further enhancement as the O_2_ ratio increases from 10 to 20%, suggesting its distinct chemistry in forming an additional carbon bond after their common rate-determining step of C–C coupling between two adsorbed CO molecules^29,31,32^. The oxygen induced enhancement of all C_2+_ products is more pronounced at −0.80 V_RHE_ (Fig. 1b), with higher than 200-fold, 100-fold, and 70-fold increases for ethylene, ethanol and acetate, respectively. As the potential becomes more negative (Fig. 1c–f), these enhancements start to reduce. At −1.0 V_RHE_, the increases of C_2+_ product formation rates become less than ten times at 20% O_2_. This could be attributed to the enhanced ORR kinetics at higher overpotentials reducing the surface coverage of its intermediates that facilitate the C–C coupling chemistry in the CO_2_RR, as well as the reduced local CO_2_ concentration caused by the increased proton consumption rate^33,34^. These mechanistic aspects will be discussed in the following sections.

C_1_ product formation rates in the CO_2_RR are also enhanced in the presence of O_2_ (Fig. 2). The onset of methane production is improved to as early as −0.75 V_RHE_ in an oxygen-containing atmosphere (Fig. 2a), while in the absence of O_2_, it is not observed until a much more negative potential of −0.95 V_RHE_ (Fig. 2d). We emphasize that an increase in the onset potential of at least 200 mV for methane formation with oxygen indicates significantly accelerated reaction kinetics, because an increase in overpotential on this scale could lead to a significant rise in reaction rates (depending on the Tafel slope) assuming the reaction is kinetically controlled near the onset potential. At −0.75 V_RHE_, the CO formation shows a slightly greater than 5-fold increase and the formate formation shows an approximately two to three times increase with 10–20% oxygen in the reaction atmosphere (Fig. 2a). As the electrode potential becomes more negatively biased (Fig. 2b–f), the O_2_ induced enhancement in C_1_ product formation rates becomes increasingly marginal and turns negative for CO and formate at −0.90 V_RHE_ and below. This trend is more pronounced at the higher (20%) O_2_ concentration. The competing hydrogen evolution reaction (HER) is also improved in the presence of O_2_ (Fig. 2), however, its degree of enhancement is not as sensitive to the applied potential. It should be noted that such significant enhancement for CO_2_RR is not observed on Cu foil electrodes with the addition of 20% O_2_ (Supplementary Figs. 4–6). This could be due to the low solubility of O_2_ in the aqueous electrolyte compared with CO_2_ (by a factor of ~26)^35^, resulting in the sluggish mass transport of O_2_ to the planer electrode surface, and thus diminishing the impact of the ORR on promoting the CO_2_RR.

### In situ surface-enhanced Raman measurements

**Fig. 3:**
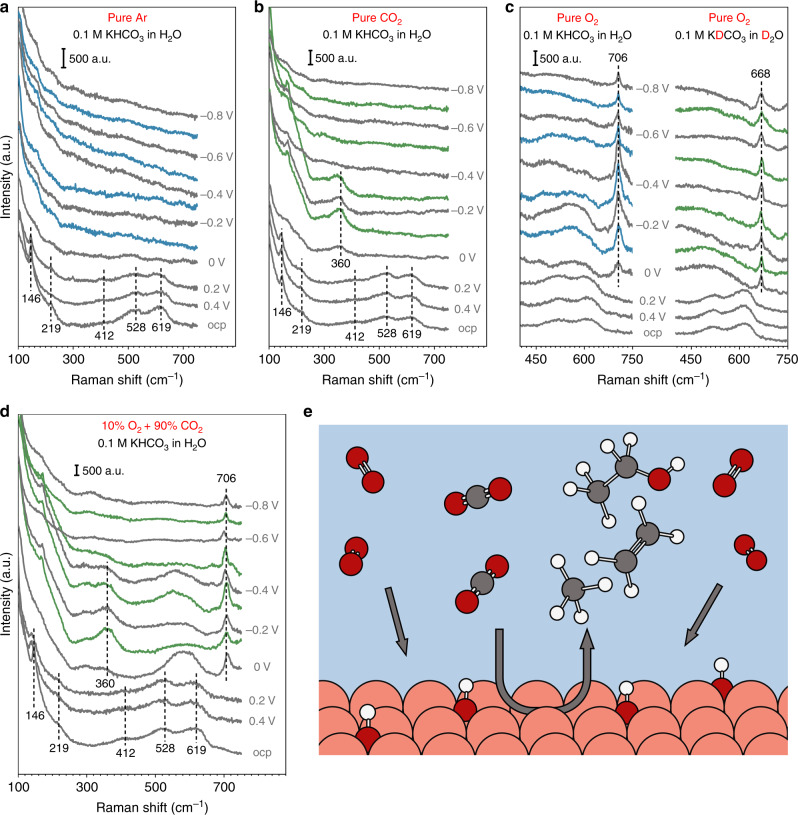
In situ surface-enhanced Raman spectroscopy. Raman spectra of Cu catalyst at electrolysis with **a** pure Ar gas feed in 0.1 M KHCO_3_/H_2_O; **b** pure CO_2_ gas feed in 0.1 M KHCO_3_/H_2_O; **c** pure O_2_ gas feed in 0.1 M KHCO_3_/H_2_O and 0.1 M KDCO_3_/D_2_O; **d** 10% O_2_ + 90% CO_2_ in 0.1 M KHCO_3_/H_2_O. **e** Schematic of CO_2_RR on Cu surface at the presence of hydroxyl groups induced by ORR.

To understand the mechanism through which co-electrolysis of CO_2_ and O_2_ boosts the CO_2_RR activity, in situ SERS is employed to identify surface species presenting during the co-electrolysis^36^. The Cu microparticle catalysts employed in this work readily exhibit the surface enhancement of Raman signals, which alleviate the need to introduce SERS-inducing particles^37,38^, and is consistent with several recent studies^36,37,39^. In the Ar atmosphere (Fig. 3a), multiple peaks at 146, 219, 412, 528, and 619 cm^−1^ are observed at the open circuit potential (OCP) and can be attributed to the surface Cu_2_O (Cu_2_O_surf_)^21,40^. These peaks decrease in intensity as the potential becomes more negative and disappear at 0 V_RHE_, due to the reduction of Cu_2_O_surf_, and no other peak is observed at more negative potentials. An additional band at 360 cm^−1^ appears after the removal of Cu_2_O_surf_ at 0 V_RHE_ in the CO_2_ atmosphere (Fig. 3b), whose assignment remains debated in the literature^36,41^, This band disappears at −0.4 V_RHE_, likely due to the conversion of adsorbed CO to other products at more negative potentials, and thus the corresponding species is unlikely to have a major impact on the CO_2_RR. A prominent band at 706 cm^−1^ appears at potentials below 0 V_RHE_ in the O_2_ atmosphere (Fig. 3c), which has been assigned to the surface hydroxyl species^21^. Control experiment in D_2_O shows a redshift of this band to 668 cm^−1^ (Fig. 3c), which confirms the binding of a protonated atom. Although the isotopic shift when switching from H_2_O to D_2_O is larger than that estimated based on the reduced mass of a [Cu-]O-H(D) bending mode (~20 cm^−1^), this is likely due to the presence of solvent. It is reasonable to assume that there is substantial hydrogen bonding between Cu-OH and the H in the surrounding water molecules, which will impact on the frequency of the Cu-OH mode. When replacing H_2_O with D_2_O, we not only replace Cu-OH with Cu-OD, but also replacing the surrounding H_2_O with D_2_O, thus the change in the reduced mass is expected to be more significant than that without the hydrogen-bonded water. The possibility of this band at 706 cm^−1^ corresponding to an adsorbed C-containing species is ruled out by the observation that this band only appear in O_2_ contained electrolyte but not in pure CO_2_ or Ar saturated electrolyte (Fig. 3). Electrolysis using ^13^CO_2_ is helpful to further confirm this argument. However, we believe the current evidences are sufficient to support the assignment of the 706 cm^−1^ band to surface hydroxyl, rather than a C-containing species. Surface hydroxyl species in the O_2_ atmosphere is likely formed during the 4-electron pathway in the conversion of O_2_ to H_2_O (Supplementary Fig. 7), as it is a known intermediate in the ORR on multiple metal surfaces^42^. Interestingly, the Cu_2_O_surf_ appears to persist to lower potentials in the O_2_ atmosphere as the broad peak at 430–650 cm^−1^ corresponding to Cu_2_O_surf_ does not completely disappear until −0.5 V_RHE_^21,36^. In the atmosphere of 10% O_2_ + 90% CO_2_, the SER spectra exhibit features of those from in both O_2_ and CO_2_ atmospheres (Fig. 3d). Importantly, the key difference between the SER spectra in the CO_2_ and O_2_ + CO_2_ atmospheres is the presence of the band corresponding to the surface hydroxyl species. This is an indication that the presence of the surface hydroxyl group, corresponding to a Raman band at 706 cm^−1^, is responsible for the distinct reactivities in the CO_2_RR in the absence and presence of O_2_ in the reaction atmosphere (Fig. 3e). SER spectra with extended spectral windows are included in the Supplementary Figs. 8 and 9 and the related peaks are discussed in Supplementary Note 1.

The CO_2_RR activity and spectroscopic results show that the formation of H_2_O_2_ is unlikely the cause of the enhanced CO_2_RR performance during the co-electrolysis with O_2_. H_2_O_2_ is a possible product of the ORR via the 2-electron pathway^43^. If produced during the co-electrolysis of CO_2_ and O_2_ on Cu, H_2_O_2_ could potentially modify the surface speciation and impact of the rate and product distribution of the CO_2_RR. Introducing up to 10 mM of H_2_O_2_ in the electrolyte of the CO_2_RR (with a pure CO_2_ feed) leads to a relatively modest change, i.e., less than 10-fold increase, in the production rates of oxygenates and hydrocarbons (Supplementary Fig. 10), which is in stark contrast with the observation of co-electrolysis with O_2_ (Figs. 1b and 2b). This is consistent with the observation that the ORR occurs mainly through the 4-electron pathway on the Cu surface (Supplementary Fig. 7) and thus the production of H_2_O_2_ is expected to be negligible. Furthermore, in situ SER spectra using electrolyte (CO_2_ saturated 0.1 M KHCO_3_) with up to 10 mM of H_2_O_2_ do not show any detectable feature corresponding to the hydroxyl species (Supplementary Fig. 11), suggesting distinct reduction mechanisms and intermediates in the ORR and the H_2_O_2_ reduction on Cu. As the H_2_O_2_ concentration in the electrolyte increases, the Raman features corresponding to Cu_2_O_surf_ persists to more negative potentials (Supplementary Fig. 11), which is expected as H_2_O_2_ is a potent oxidant. Importantly, the lack of the surface hydroxyl group and the modest change in the CO_2_RR activity with added H_2_O_2_ support the hypothesis that the surface hydroxyl group formed during the co-electrolysis is responsible for the enhanced CO_2_RR performance.

### Density functional theory calculations

**Fig. 4:**
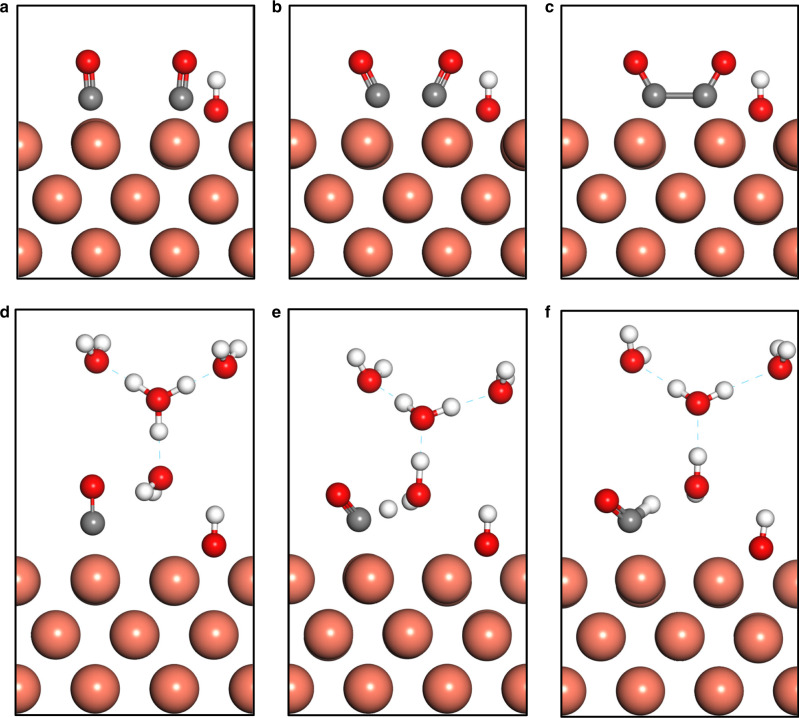
Computational model. Representative structures of *CO dimerization at initial states (**a**), transition states (**b**), and final states (**c**). Representative structures of *CO hydrogenation at initial states (**d**), transition states (**e**), and final states (**f**).

**Fig. 5:**
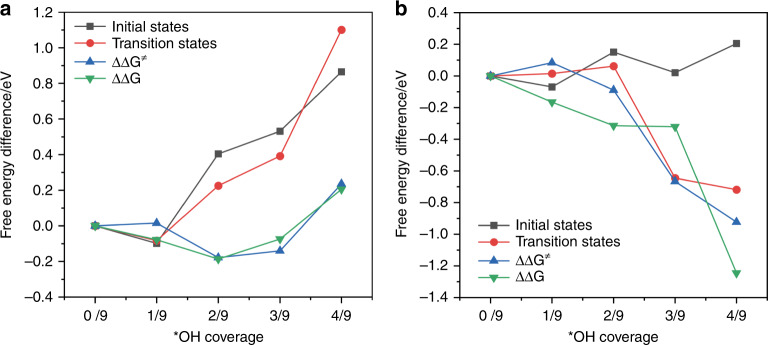
Free energy diagram. **a** *CO dimerization. **b** *CO hydrogenation. The binding free energies for initial states and transition states at different *OH coverage are calculated with reference to a Cu slab with specific numbers of *OH, gas phase CO, H_2_, and liquid H_2_O molecules. The potential is −0.5 V_SHE_ (i.e., equivalent to −0.9 V_RHE_). ΔΔ*G* (ΔΔ*G*^**≠**^) is defined as the reaction-free energy (free energy barrier) at certain *OH coverage referenced to the value obtained at zero *OH coverage (see “Methods” section for computational details).

To further evaluate the hypothesized beneficial effect of the hydroxyl group on the CO_2_RR, potential energy landscapes of key reaction steps are estimated via density functional theory calculations. The free energy profiles of the rate-determining steps (RDS) in the formation of C_2+_ products, i.e., *CO dimerization^29,31,44,45^ and methane, i.e., *CO hydrogenation^29,44,46^, are calculated at different *OH coverage on the Cu(100) facet, with the representative model structures and results shown in Figs. 4 and 5, respectively^31,45,46^. −0.5 V_SHE_ (i.e., equivalent to −0.9 V_RHE_) is chosen as the potential in our calculations. Cu(100) facet is employed for the computational study. Cu(100) and Cu(111) are the most exposed facets on polycrystalline Cu surface because of their low surface energies^47^. It has been shown that polycrystalline Cu surface undergoes surface reconstruction to form Cu(100) under the CO_2_RR conditions, which is also consistent with experiments showing that polycrystalline Cu leads to a product distribution similar to that on Cu(100)^48^. Therefore, Cu(100) facet is commonly used as a representative model surface for obtaining theoretical insights into experimental studies based on polycrystalline Cu^46,49^. Our computational model also consists hollow-site adsorbed *OH with different coverage. This is because *OH is most stable on hollow sites at the negative potentials described by the VASPsol model. The vibrational modes for hollow-site *OH in the vicinity of *CO were calculated at the initial states of *CO dimerization and *CO hydrogenation (Supplementary Tables 1 and 2). We employ a well-established finite difference method to calculate the vibrational modes of the *OH adsorbate^49,50^. The calculated wavenumbers of vibration modes of *OH are compared with the Raman band observed experimentally. The results show good agreement with the observed Raman band of 706 cm^−1^ in the in situ SERS experiments, which supports the representativeness of the computational model employed in this work. Due to the limitations of DFT calculations, other characters such as band width and intensity, cannot be accurately predicted and thus are not discussed.

Both the activation barrier and the free energy change for *CO dimerization decrease as the *OH coverage increases from 0 to 3/9 but rebound with further increase in *OH coverage (Fig. 5a). Both the initial state and transition state become less stable as the *OH coverage increases. This could be attributed to the increase of oxidation state of surface Cu as revealed by Bader charge analysis (Supplementary Fig. 12), which weakens the π-back-donation from the Cu to the anti-bonding orbital of the C≡O bond^51,52^. The magnitude of destabilization of the initial state is greater than that of the transition state when the *OH coverage is at or below 3/9, leading to the decrease of the reaction barrier. This is consistent with the observed enhancement of C_2+_ product formation rate with ≤20% O_2_ in the gas feed (Fig. 1). As the *OH coverage increases beyond 3/9, both the activation barrier and the free energy change for *CO dimerization increase substantially, which is likely due to the repulsive interaction with the excess *OH groups nearby.

For *CO hydrogenation to *CHO, both activation barrier and free energy change decrease substantially when *OH coverage increases from 1/9 to 4/9 (Fig. 5b). Notably, these decreases are much more significant than those in *CO dimerization, which agrees with the significant anodic shift of methane onset potential in the presence of the concurrent ORR (Fig. 2). The energies of initial structures fluctuate slightly within an energy range of 0.2 eV when *OH coverage increases, however, the transition state of *CO hydrogenation is stabilized under same conditions. We speculate that this is likely due to the transition state forms additional hydrogen bonds with *OH groups at higher coverage. The stabilization of reactant by forming hydrogen bonds with *OH has been reported in a previous work^53^.

## Discussion

A key difficulty in elucidating the role of oxidized Cu species on the surface in promoting the CO_2_RR is their poor stability under the reducing condition, which renders their composition and structure ambiguous. This stems from the fact that these species are prepared at a more oxidizing atmosphere, thermochemically or electrochemically, and then brought to a reducing environment. This challenge is circumvented by providing a continuous supply of oxidant in the CO_2_RR, i.e., co-electrocatalysis of CO_2_ and O_2_, to stabilize the surface oxidized Cu species, i.e., surface hydroxyl group. Co-electrolysis could also be viewed as coupled reactions, with the ORR supplying reactive oxygen species to form and replenish the surface hydroxyl species vital to the enhancement of the CO_2_RR. Control experiments with H_2_O_2_ show that it is not H_2_O_2_ or intermediates of its reduction because neither the CO_2_RR activity nor the spectral feature with H_2_O_2_ resembles that in the co-electrolysis at otherwise identical conditions. The presence of H_2_O_2_ in the electrolyte makes Cu_2_O_surf_ more resistant to negative potentials (Supplementary Fig. 11), even to −0.8 V_RHE_ at 10 mM of H_2_O_2_. The fact that H_2_O_2_ does not significantly promote the CO_2_RR shows that the CO_2_RR performance is highly sensitive to the nature of the surface species. As different oxidants lead to different surface species, results reported in this work establish a paradigm for enhancing the CO_2_RR by co-electrolyzing CO_2_ with an oxidant. The nature and concentration of oxidants could be designed to tune the nature and coverage of surface oxidized Cu species. This hypothesis is supported by the contrasting results of using H_2_O_2_ and O_2_ oxidant, as well as different partial pressure of O_2_. Higher partial pressure of O_2_ in the feed (up to 20%) is expected to increase the surface coverage of the adsorbed hydroxyl species, which is generally beneficial toward the improvement of production rates of oxygenates and hydrocarbons. The diminishing impact of O_2_ on the CO_2_RR at potentials below −0.9 V_RHE_ could be attributed to at least two factors: (1) Reduction of surface coverage of the hydroxyl species due to its facile reduction at such negative potentials; unfortunately, excessive bubble formation prevents spectroscopic investigations at these potentials. (2) Reduction in the local CO_2_ concentration due to the accelerated CO_2_RR and increased alkalinity caused by the fast proton consumption from both the CO_2_RR and the HER^33,34^. The increase of electrolyte pH has been demonstrated to not affect C_2+_ product formations because the RDS does not involve any proton transfer^28,44,54,55^. However, higher pH is known to increase the hydrogen binding energy (HBE)^56,57^, which accelerates the HER on Cu^58^. This is consistent with the observed enhancement in the HER rate with the addition of O_2_ (Fig. 2). The enhancement of HER rate is relatively insensitive to the applied potential. This is likely due to the interfacial pH at the cathode does not change significantly with electrolysis potentials because the ORR is severely mass-transport limited at such large overpotentials (>1.8 V). In addition to enhancing the CO_2_RR rates, the introduction of O_2_ also significantly reduces the Faradaic efficiency (FE) of CO_2_RR products (Supplementary Fig. 3b, c), presumably by introducing a competing reaction, i.e., the ORR. We note that at higher O_2_ concentrations than 20%, a limited increase in the production rate of the CO_2_RR with elevated ORR activity and reduced Faradaic efficiency for the CO_2_RR are expected based on current results. Reliable measurements of electrolysis at higher O_2_ concentrations are challenging due to the instability of the electrode at high current densities (over 100 mA cm^−^^2^) in an H-type electrochemical cell. Systematic investigations of the impact of O_2_ partial pressure on the CO_2_RR activity require enhanced mass transport which could be more easily accomplished in flow cell configurations^59–62^, and are outside the scope of this proof-of-concept work. It is important to note that any practical CO_2_ source contains large quantities of O_2_ can be utilized, e.g., flue gas from power plants or air, so CO_2_RR systems that are tolerant to or enhanced by a low concentration of O_2_ in the feed could significantly reduce the separation cost. In the meantime, co-electrolysis of other oxidants, e.g., peroxyacetic acid, ammonium persulfate, could be a fruitful strategy to further improve the rate and efficiency of CO_2_RR.

## Methods

### Materials

The polycrystalline Cu powder (−625 mesh, APS 0.50–1.5 micron, 99% metal basis) is purchased from Alfa Aesar. Cu foil (0.1 mm thick, 99.9999% metal basis) is purchased from Alfa Aesar. Potassium carbonate (99.997% trace metals basis) is purchased from Alfa Aesar. Chelex 100 sodium form is purchased from Sigma-Aldrich. Isopropanol (99.999% trace metal basis) is purchased from Sigma-Aldrich. Dimethyl sulfoxide (≥99.9%) is purchased from Alfa Aesar. Deuterium oxide (99.9 atom% D) is purchased from Sigma-Aldrich. Nafion solution (5 wt%) is purchased from Sigma-Aldrich. Phosphoric acid (ACS reagent, ≥85 wt% in H_2_O) is purchased from Sigma-Aldrich. Graphite rod (99.995% trace metals basis) is purchased from Sigma-Aldrich. Sigracet 39 BC carbon fiber paper is purchased from Fuel Cell Store. Carbon Dioxide (99.999%), oxygen (99.999%), and argon (99.999%) are purchased from Air Liquide. All electrolyte solutions are prepared using Milli-Q water (18.2 MΩ cm).

### Electrode preparation

To prepare the polycrystalline Cu power electrode, an ink solution is first prepared by mixing 1 mg Cu powder and 1 mL isopropanol followed by sonicating for 20 min. 900 μL of ink solution is dropcasted onto the microporous layer of a 3 × 3 cm^2^ Sigracet 39 BC carbon fiber paper. After drying in air, 180 μL of 2.5 wt% Nafion solution (diluted with isopropanol) is uniformly deposited onto the catalyst surface. The catalyst is dried in the air again and transferred into a vacuum box to thoroughly remove the residual solvent. The catalyst is then cut into small pieces with a size of 0.6 × 1.5 cm^2^ and a nickel wire current collector is attached to a piece of the catalyst using silver epoxy. To prepare the electropolished Cu foil electrode, a nickel wire current collector is welded to a piece of Cu foil. The electrode is then electropolished in 85% phosphoric acid at 2.1 V versus a graphite rod electrode for 5 min followed by rinsing with Mill-Q water (18.2 MΩ cm). A fresh working electrode is used for each electrochemical measurement.

### Electrochemical measurements

All electrochemical measurements are conducted in a custom designed gastight two-compartment electrochemical cell fabricated by Adams & Chittenden Scientific Glass with three-electrode configuration. A graphite rod is used as counter electrode and a Ag/AgCl (3.0 M, BASi) electrode is used as reference electrode. The two chambers of the electrochemical cell are separated by anion-exchange membrane (Selemion AMV, AGC Inc.). The electrolyte used for all electrochemical measurements is CO_2_-saturated 0.1 M KHCO_3_ solution with a pH of 6.8, which is prepared by purging 0.05 M K_2_CO_3_ solution with CO_2_ overnight, and the electrolyte is purified with Chelex before electrolysis.

All electrochemical measurements are conducted using a Gamry Reference 600+ Potentiostat and the measured potential is converted to RHE scale using the following formula: *E*_RHE_ = *E*_Ag/AgCl_ + 0.210 + 0.05916 × pH (in volts), where the standard value for the Ag/AgCl reference electrode is calibrated using a homemade standard hydrogen electrode. The uncompensated resistance (*R*_u_) is measured by potentiostatic electrochemical impedance spectroscopy and 100% of *R*_u_ is compensated by the potentiostat during electrolysis. All electrodes are pretreated in Ar-purged electrolyte at −1 V_RHE_ for 5 min to stabilize surface conditions. The gas flow is then switched to reactant gas (pure CO_2_ or 10% O_2_ + 90% CO_2_ or 20% O_2_ + 80% CO_2_) and the system is purged for 15 min prior to a 1 h electrolysis. All gases are directly delivered into electrolyte through a gas dispersion frit at a total flow rate of 10 mL/min. The flow rate is controlled by mass flow controllers (MKS Instruments Inc.) and calibrated by an ADM flow meter (Agilent Technologies). The O_2_ + CO_2_ mixture is prepared by mixing CO_2_ and O_2_ at desired ratio using mass flow controllers at a total flow rate of 10 mL/min. The outlet flow of the electrochemical cell is directly vented to the sample loop of a gas chromatograph (Agilent 7890B) for product quantification.

### Product quantification

The gaseous products are quantified every 20 min using a gas chromatograph (GC) (Agilent 7890B) equipped with a ShinCarbon ST column and a HayeSep Q column. A thermal conductivity detector is used to quantify H_2_ and a flame ionization detector with a methanizer is used to quantify CO, CH_4_, and C_2_H_4_. Ar is used as carrier gas. The analysis results of three GC analyses during the 1 h electrolysis are averaged. The liquid products are quantified using ^1^H NMR on a Bruker AVIII 400 MHz NMR spectrometer after the whole electrolysis process. The NMR sample is prepared by mixing 500 μL of the electrolyte collected after electrolysis with 100 μL of internal standard solution (1.67 ppm (m/m) dimethyl sulfoxide in D_2_O). The water signal is suppressed using the presaturation method.

### Determination of ORR electron transfer number

The ORR electron transfer number of polycrystalline Cu powder catalyst is determined using rotating disk electrode (RDE) measurement and Koutecký–Levich equation method. To prepare the electrode for RDE measurement, an ink solution is first prepared by mixing 5 mg Cu powder, 25 μL Nafion solution and 1 mL 2-propanol followed by sonicating for 30 min in an ice cold water bath. A 20 μL ink is then dropcasted onto a glassy carbon rotating disk electrode (0.5 cm diameter, Gamry). A graphite rod is used as counter electrode and a double junction Ag/AgCl electrode (3.0 M) is used as reference electrode. The experiments are conducted using a Gamry RDE 710 system and the electrolyte is 0.1 M KHCO_3_ solution. The cyclic voltammogram (CV) is firstly taken under Ar at a scan rate of 10 mV/s with a rotation speed of 2500 rpm prior to ORR measurement. The electrolyte is then bubbled with O_2_ for at least 20 min to saturate the electrolyte. CVs in O_2_ atmosphere are recorded at various rotation speed (400, 625, 900, 1225, 1600, 2025, and 2500 rpm) at a scan rate of 10 mV/s.

### In situ Raman spectroscopy measurements

In situ Raman spectroscopy measurements are conducted using a custom-made three-electrode electrochemical cell in H-cell configuration that consists two compartments and is separated by a piece of Nafion ion exchange membrane (IEM, Nafion 211, Fuel Cell Store) (Supplementary Fig. 13). The three-electrode system is made up of a Cu working electrode that is identical to the ones in reactivity measurement, a Ag/AgCl reference electrode (3.0 M NaCl, BASi) and a graphite counter electrode. A potentiostat (VersaSTAT, Princeton) is used to perform electrolysis. Electrolyte saturated with desired gas is delivered into the cell using a HPLC pump (GP50 Gradient Pump, Dionex) to achieve similar mass transport as in magnetically stirred reactivity cell. The Raman spectroscopy measurements are carried out using LabRAM HR Evolution microscope (Horiba Jobin Yvon) equipped with a 632.8 nm He-Ne laser, a ×50 objective (NA = 0.55), and a CCD detector. The filter is set to be 50% to keep a low laser intensity to avoid any irradiation-induced modifications of Cu surface. The acquisition time is 20 s for each spectrum with the accumulation times of 2.

### Computational details

The Cu(100) metal slab (3 × 3) consisting of three layers with the bottom layer fixed in its bulk position is employed to simulate the surface of Cu. The total energy of the slab with different adsorbates are calculated using DFT with the Perdew–Burke–Ernzerhof exchange-correlation functional in plane-wave pseudopotentials, as implemented in the Vienna ab initio Simulation Package (VASP)^63^. The empirical D2 approach as implemented in VASP is employed to describe the van der Waals interactions. All calculated energy values are extrapolated to *k*_B_*T* = 0. A Monkhorst–Pack k-point net of 3 × 3 × 1 is chosen to sample the reciprocal space. A vacuum of 25 Å is introduced to each side to avoid interactions between successive metal slabs.

VASPsol model is employed to establish the electrochemical interface^64,65^. In this model, the Fermi energy is adjusted to a target value by changing the number of electrons in the system during each step of the geometry optimization, which keeps the work function and electrode potential constant in the calculations. Then the linear Poisson–Boltzmann implicit solvation model with a Debye screening length of 3.0 Å is used to neutralize the non-zero charge in the simulation cell and simulate water and the electrolyte, allowing for a more realistic description of the electrochemical double layer. A detailed description of this approach has been provided in our previous work^66–68^.

The transition state for each reaction is first approached using the nudged elastic band (NEB) method in the neutral state. Forces on the climbing image are converged to <0.02 eV/Å. The plane-wave cutoff, smearing parameter and functional, and calculator parameters are the same as those used in slab geometry optimizations. Structures obtained from NEB are employed to generate the input structure and orientation for the dimer calculation. The force of the dimer calculation is converged to <0.1 eV/Å to accurately locate the saddle point, i.e., the transition state. After that, the free energy of transition state is calculated under constant potential. In the calculations for *CO hydrogenation, the required H^+^/*e*^−^ pair for reduction is assumed to originate from the aqueous solution and the electrode. Its free energy is estimated based on the computational hydrogen electrode model^69^. In addition to the implicit Poisson–Boltzmann solvation model, all the stationary points along the hydrogenation step are solvated by four explicit water molecules to provide a better description of the solvent. All possible structures, including different adsorption sites of *CO and *OH, relative position between *CO and *OH, etc., are investigated at all *OH coverage. The most stable structures are employed to investigate the impact of *OH adsorbate to the RDS of C_2+_ product formations and CH_4_ formation (Supplementary Figs. 14–17).

The free energies of the slab systems were calculated as follows:

1 $$G = E_{{\mathrm{elec}}}^{{\mathrm{solv}}} + {\mathrm{ZPVE}} + H_{{\mathrm{vib}}} - {\mathrm{TS}}_{{\mathrm{vib}}},$$

where $E_{{\mathrm{elec}}}^{{\mathrm{solv}}}$ was the electronic energy of the system calculated from VASPsol. We treated all degrees of freedom of the adsorbates as vibrational and neglect the contribution of vibrations of the slab. The vibrational frequencies (*ν*) were evaluated by calculating the partial Hessian matrix through the finite difference method. Unusually low vibrational modes (<50 cm^−1^) were reset to 50 cm^−1^ to avoid unphysically large entropy contributions. Based on the calculated vibrational frequencies, we calculated the zero-point vibrational energy (ZPVE), vibrational contributions to the internal energy (*H*_vib_) and entropy (*S*_vib_) at 298 K as follows:

2 $${\mathrm{ZPVE}} = \mathop {\sum}\limits_\nu {\frac{{h\nu }}{2}},$$

3 $$H_{{\mathrm{vib}}} = \mathop {\sum}\limits_\nu {\frac{{h\nu }}{{e^{h\nu /k_{\mathrm{B}}T} - 1}}},$$

4 $$S_{{\mathrm{vib}}} = k_{\mathrm{B}}\mathop {\sum}\limits_\nu {\left[ {\frac{{h\nu }}{{k_{\mathrm{B}}T\left( {e^{h\nu /k_{\mathrm{B}}T} - 1} \right)}} - {\mathrm{ln}}\left( {1 - e^{ - h\nu /k_{\mathrm{B}}T}} \right)} \right]}.$$

The free energies of the molecules were determined as follows:

5 $$G = E_{{\mathrm{elec}}}^{{\mathrm{solv}}} + {\mathrm{ZPVE}} + \left( {\frac{n}{2} + 1} \right)k_{\mathrm{B}}T + H_{{\mathrm{vib}}} - T(S_{{\mathrm{vib}}} + S_{{\mathrm{trans}}} + S_{{\mathrm{rot}}}),$$

where *n* is 6 for non-linear molecules and 5 for linear molecules. ZPVE was calculated as shown above. *H*_vib_, *S*_vib_, *S*_rot_, and *S*_trans_ were obtained from Jaguar using the PBE/6-31G* basis set. The correction terms of all free energies are provided in Supplementary Table 3.

For *CO dimerization, the adsorption free energy of each state was calculated as:

6 $$G_{{\mathrm{abs}}}^{{\mathrm{IS}}}\left( {{{n}}/9\,^\ast {\mathrm{OH}}\,{\mathrm{coverage}}} \right) = G_{{\mathrm{IS}}}\left( {{{n}}/9\,^\ast {\mathrm{OH}}\,{\mathrm{coverage}}} \right) - G_{{\mathrm{Cu}} + {{n}}^\ast {\mathrm{OH}}}-2G_{^\ast {\mathrm{CO}}},$$

7 $$G_{{\mathrm{abs}}}^{{\mathrm{TS}}}\left( {{{n}}/9\,^\ast {\mathrm{OH}}\,{\mathrm{coverage}}} \right) = G_{{\mathrm{TS}}}\left( {{{n}}/9\,^\ast {\mathrm{OH}}\,{\mathrm{coverage}}} \right) - G_{{\mathrm{Cu}} + {{n}}^\ast {\mathrm{OH}}}-2G_{^\ast {\mathrm{CO}}},$$

8 $$G_{{\mathrm{abs}}}^{{\mathrm{FS}}}\left( {{{n}}/9\,^\ast {\mathrm{OH}}\,{\mathrm{coverage}}} \right) = G_{{\mathrm{FS}}}\left( {{{n}}/9\,^\ast {\mathrm{OH}}\,{\mathrm{coverage}}} \right) - G_{{\mathrm{Cu}} + {{n}}^\ast {\mathrm{OH}}}-2G_{^\ast {\mathrm{CO}}}.$$

For *CO hydrogenation, the adsorption free energy of each state was calculated as:

9 $$G_{{\mathrm{abs}}}^{{\mathrm{IS}}}\left( {{{n}}/9\,^\ast {\mathrm{OH}}\,{\mathrm{coverage}}} \right) = G_{{\mathrm{IS}}}\left( {{{n}}/9\,^\ast {\mathrm{OH}}\,{\mathrm{coverage}}} \right) - G_{{\mathrm{Cu}} + {{n}}^\ast {\mathrm{OH}}}-G_{^\ast {\mathrm{CO}}}-4G_{{\mathrm{H}}_2{\mathrm{O}}},$$

10 $$G_{{\mathrm{abs}}}^{{\mathrm{TS}}}\left( {{{n}}/9\,^\ast {\mathrm{OH}}\,{\mathrm{coverage}}} \right) = \, G_{{\mathrm{TS}}}\left( {{{n}}/9\,^\ast {\mathrm{OH}}\,{\mathrm{coverage}}} \right) - G_{{\mathrm{Cu}} + {{n}}^\ast {\mathrm{OH}}}\\ \,-\, G_{^\ast {\mathrm{CO}}}-4G_{{\mathrm{H}}_2{\mathrm{O}}}-1/2G_{{\mathrm{H}}_2},$$

11 $$G_{{\mathrm{abs}}}^{{\mathrm{FS}}}\left( {{{n}}/9\,^\ast {\mathrm{OH}}\,{\mathrm{coverage}}} \right) = \, G_{{\mathrm{FS}}}\left( {{{n}}/9\,^\ast {\mathrm{OH}}\,{\mathrm{coverage}}} \right)\\ \,-\, G_{{\mathrm{Cu}} + {{n}}^\ast {\mathrm{OH}}}-G_{^\ast {\mathrm{CO}}}-4G_{{\mathrm{H}}2{\mathrm{O}}}-1/2G_{{\mathrm{H}}_2}.$$

For the results presented in Fig. 5:

12 $${\Delta}G_{{\mathrm{abs}}}^{{\mathrm{IS}}}\left( {{{n}}/9\,^\ast {\mathrm{OH}}\,{\mathrm{coverage}}} \right) = G_{{\mathrm{abs}}}^{{\mathrm{IS}}}\left( {{{n}}/9\,^\ast {\mathrm{OH}}\,{\mathrm{coverage}}} \right) - G_{{\mathrm{abs}}}^{{\mathrm{IS}}}\left( {{\mathrm{zero}}\,{\mathrm{coverage}}} \right),$$

13 $${\Delta}G_{{\mathrm{abs}}}^{{\mathrm{TS}}}\left( {{{n}}/9\,^\ast {\mathrm{OH}}\,{\mathrm{coverage}}} \right) = G_{{\mathrm{abs}}}^{{\mathrm{TS}}}\left( {{{n}}/9\,^\ast {\mathrm{OH}}\,{\mathrm{coverage}}} \right) - G_{{\mathrm{abs}}}^{{\mathrm{TS}}}\left( {{\mathrm{zero}}\,{\mathrm{coverage}}} \right),$$

14 $${\Delta}{\Delta}G = {\Delta}G\left( {{{n}}/9\,^\ast {\mathrm{OH}}\,{\mathrm{coverage}}} \right) - {\Delta}G\left( {{\mathrm{zero}}\,^\ast {\mathrm{OH}}\,{\mathrm{coverage}}} \right),$$

15 $${\Delta}{\Delta}G^ \ne = {\Delta}G^ \ne \left( {{{n}}/9\,^\ast {\mathrm{OH}}\,{\mathrm{coverage}}} \right) - {\Delta}G^ \ne \left( {{\mathrm{zero}}\,^\ast {\mathrm{OH}}\,{\mathrm{coverage}}} \right),$$

where

16 $${\Delta}G\left( {{{n}}/9\,^\ast {\mathrm{OH}}\,{\mathrm{coverage}}} \right) = G_{{\mathrm{abs}}}^{{\mathrm{FS}}}\left( {{{n}}/9\,^\ast {\mathrm{OH}}\,{\mathrm{coverage}}} \right) - G_{{\mathrm{abs}}}^{{\mathrm{IS}}}\left( {{{n}}/9\,^\ast {\mathrm{OH}}\,{\mathrm{coverage}}} \right),$$

17 $${\Delta}G^ \ne \left( {{{n}}/9\,^\ast {\mathrm{OH}}\,{\mathrm{coverage}}} \right) = G_{{\mathrm{abs}}}^{{\mathrm{TS}}}\left( {{{n}}/9\,^\ast {\mathrm{OH}}\,{\mathrm{coverage}}} \right) - G_{{\mathrm{abs}}}^{{\mathrm{IS}}}\left( {{{n}}/9\,^\ast {\mathrm{OH}}\,{\mathrm{coverage}}} \right),$$

are the free energy change and free energy barrier for the reaction (*CO dimerization or *CO hydrogenation) at certain *OH coverage, respectively.

### Physical characterization

The microstructure of the Cu catalyst is characterized by field emission scanning electron microscope (Merlin FE-SEM, Zeiss). Powder X-ray diffraction pattern is collected using a Rigaku MiniFlex 600 with Cu Kα radiation. X-ray photoelectron spectroscopy measurements are conducted on a PHI Quantera II and CasaXPS software (Casa Software Ltd., UK) is used to analyze the resulting spectra.

## Supplementary information

Supplementary Information

## Data Availability

The data that support the findings of this study are available from the corresponding author upon request.
