# Supplementary material for: Oxygen induced promotion of electrochemical reduction of CO2 via co-electrolysis
Source: Nat Commun. 2020 Jul 31;11:3844. doi: 10.1038/s41467-020-17690-8 (PMC7395777; doi:10.1038/s41467-020-17690-8)
Supplement: Supplementary file 1 — Supplementary Information [file 41467_2020_17690_MOESM1_ESM.pdf]

Supplementary Information for

He *et al*., Oxygen induced promotion of electrochemical reduction of CO_2 via co-electrolysis

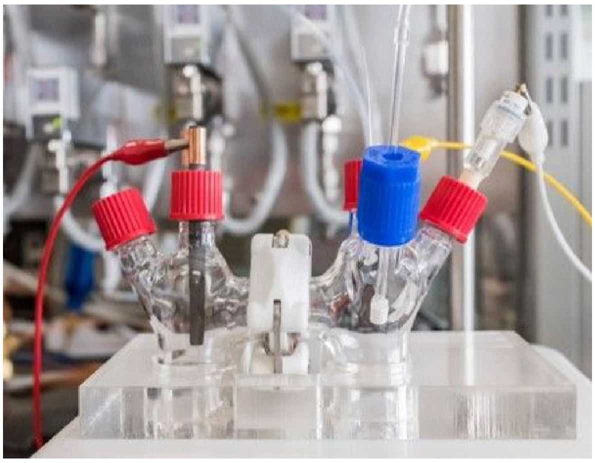

Supplementary Figure 1. Image of the three-electrode H-type electrochemical cell employed for electrolysis

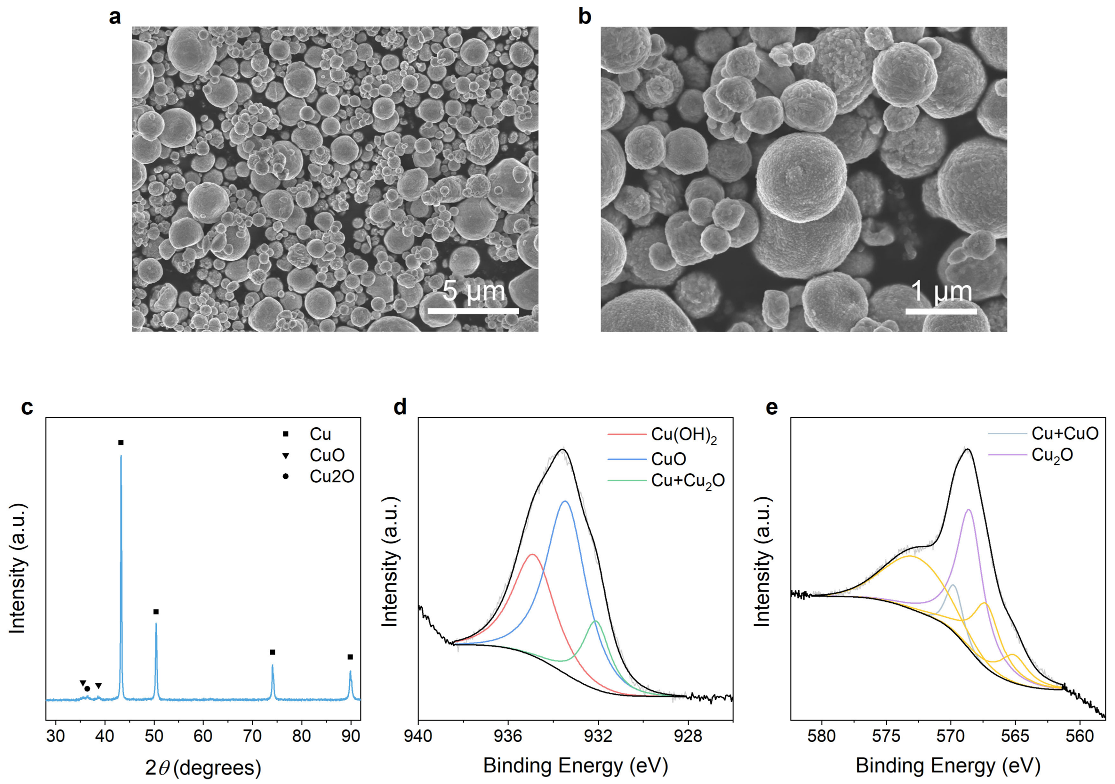

Supplementary Figure 2. Physical characterizations of the polycrystalline Cu powder electrode. (a-b) SEM images at different magnifications. (c) Powder X-ray diffraction patterns. X-ray photoelectron spectra of (d) the Cu $2p_{3/2}$ peaks and (e) the Cu LMM region. The three additional peaks (yellow) in the Cu LMM spectra located at approximately 572.8, 567.0 and 565.1 eV only represent different transition states[1, 2].

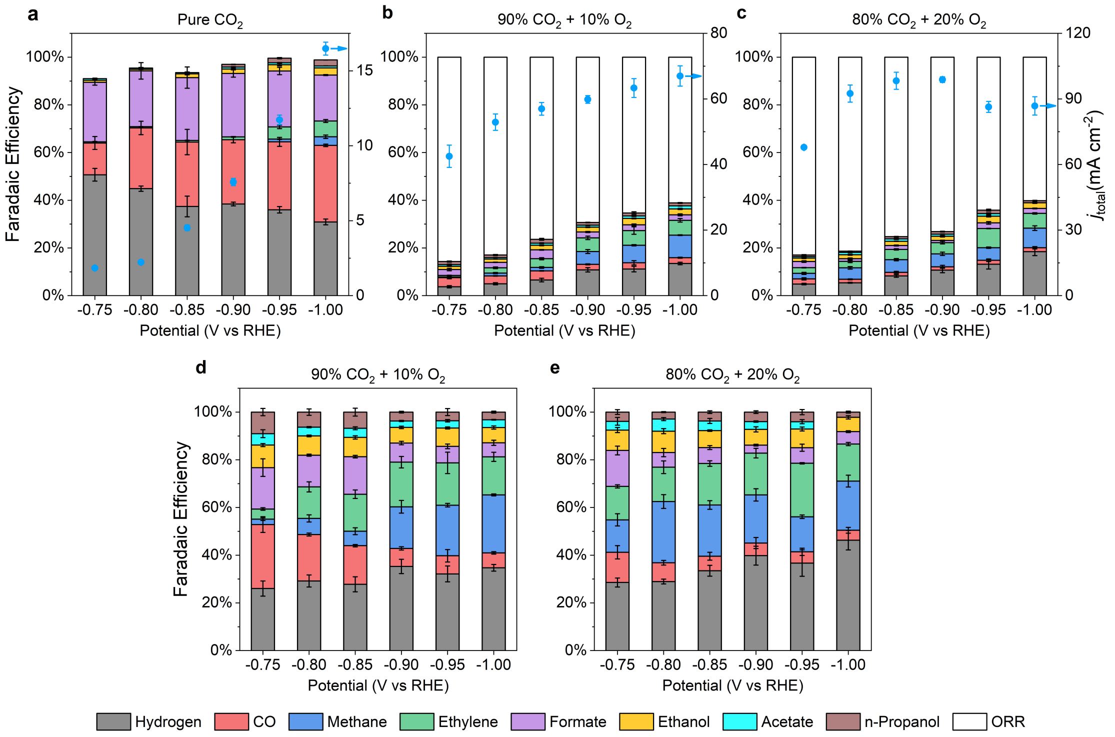

Supplementary Figure 3. Total current densities and Faradaic efficiencies of the polycrystalline Cu powder electrode measured at (a) pure CO_2, (b) 90% CO_2 + 10% O_2, (c) 80% CO_2 + 20% O_2 and normalized Faradaic efficiencies of the polycrystalline Cu powder electrode measured at (d) 90% CO_2 + 10% O_2, (e) 80% CO_2 + 20% O_2. The error bars represent the standard deviation from at least three independent measurements. The corresponding data are provided in Supplementary Table 4 and 5.

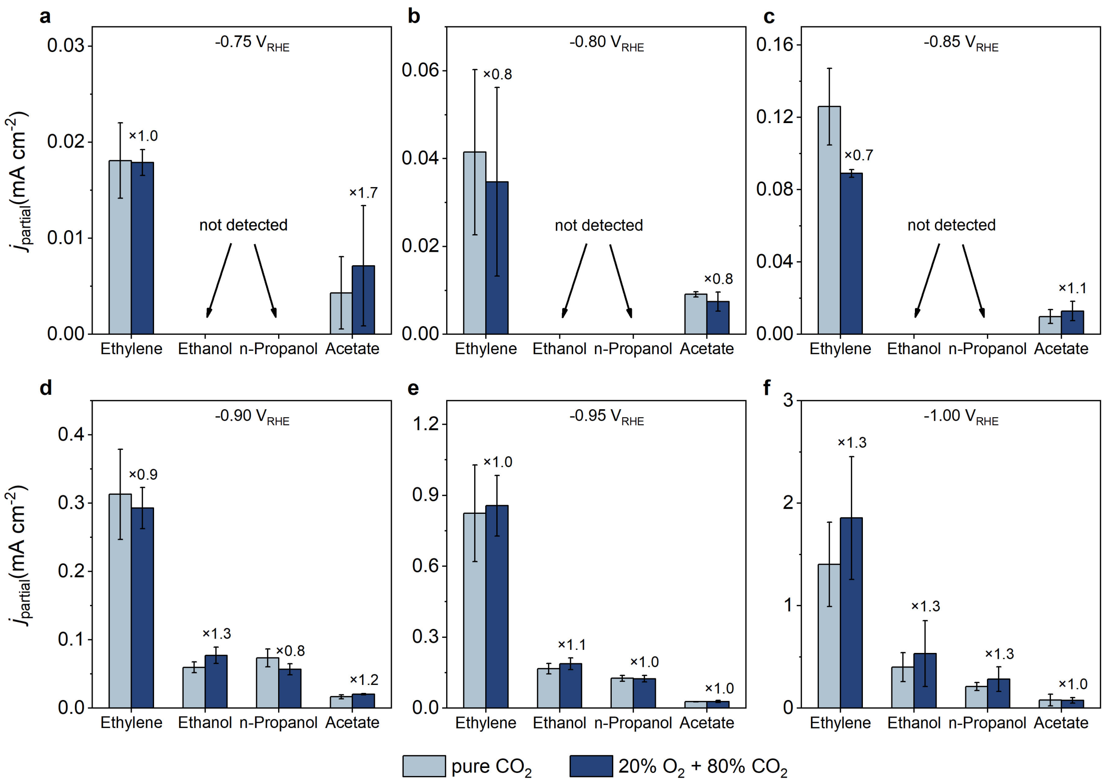

Supplementary Figure 4. Comparison of C_{2+} product formations on the electropolished Cu foil electrode. The partial current densities of C_{2+} products measured at 100% CO_2 and 20% O_2 +80% CO_2 are compared at different potentials of (a) -0.75 V_{RHE}, (b) -0.80 V_{RHE}, (c) -0.85 V_{RHE}, (d) -0.90 $V_{RHE,}$ (e) -0.95 V_{RHE} and (f) -1.0 V_{RHE}. The numbers stand for the enhancement relative to the rates at pure CO_2. The error bars represent the standard deviation from at least three independent measurements.

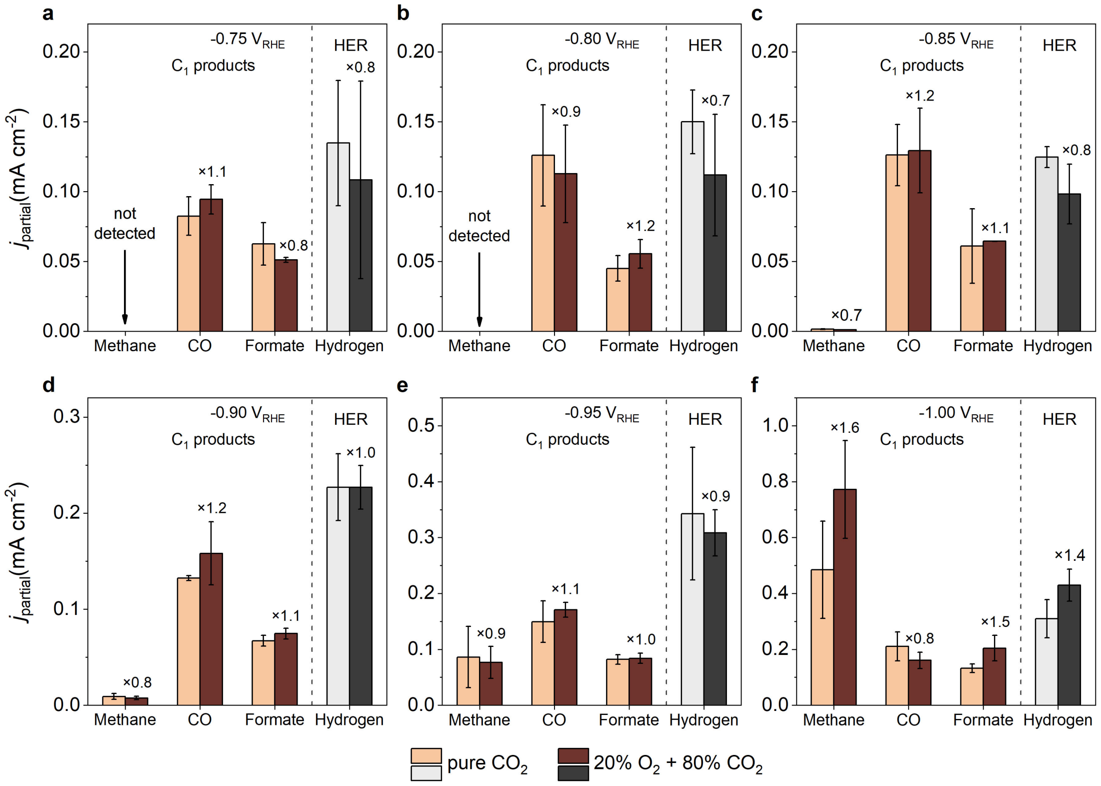

Supplementary Figure 5. Comparison of C_1 product and H_2 formations on the electropolished Cu foil electrode. The partial current densities of C_1 products and H_2 measured at 100% CO_2 and 20% O_2 +80% CO_2 are compared at different potentials of (a) -0.75 V_{RHE}, (b) -0.80 V_{RHE}, (c) -0.85 V_{RHE}, (d) -0.90 $V_{RHE,}$ (e) -0.95 V_{RHE} and (f) -1.0 V_{RHE}. The numbers stand for the enhancement relative to the rates at pure CO_2. The error bars represent the standard deviation from at least three independent measurements.

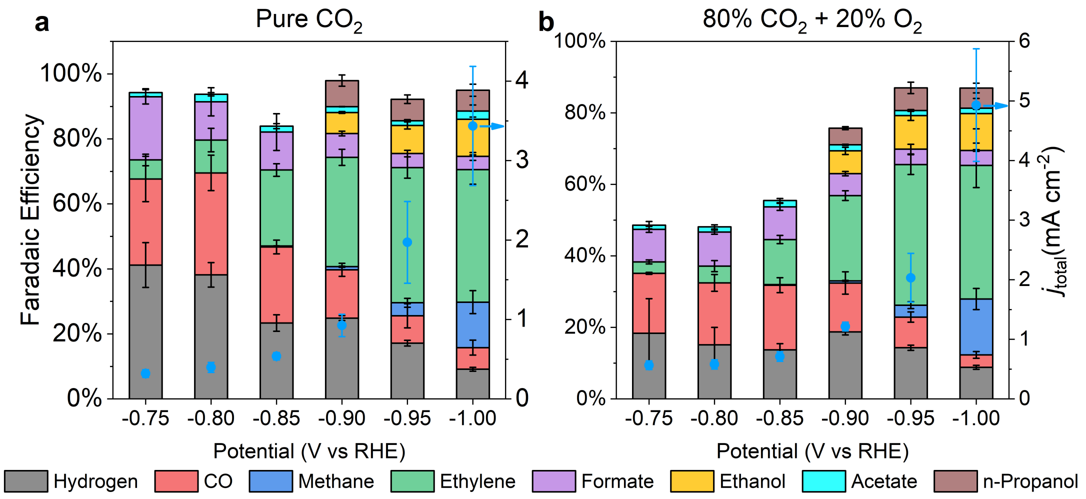

Supplementary Figure 6. Total current densities and Faradaic efficiencies of the electropolished Cu foil electrode measured at (a) pure CO_2, (b) 80% CO_2 + 20% O_2. The error bars represent the standard deviation from at least three independent measurements.

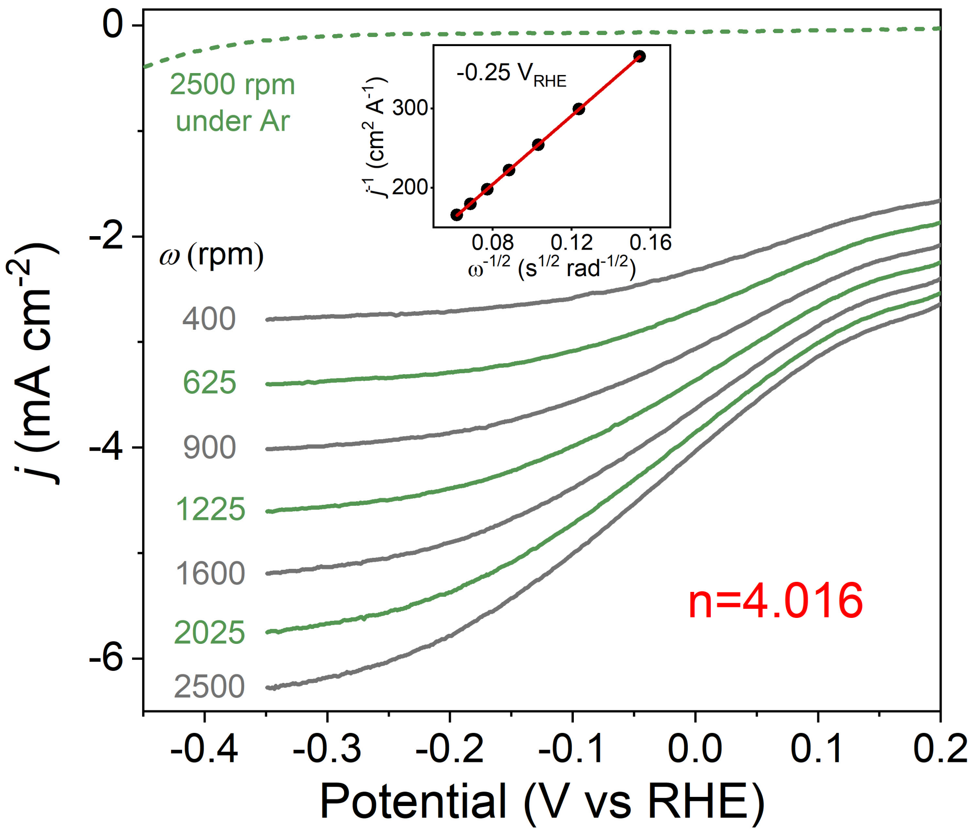

Supplementary Figure 7. Cyclic voltammetry curves of polycrystalline Cu powder catalyst recorded in 0.1 M $KHCO_3$ saturated with Ar (dashed line) and O_2 (solid lines) at a scan rate of 10 mV s^{-1} at various rotation speed. The insert shows the corresponding Koutecky–Levich plot at -0.25 V_{RHE}. The ORR electron transfer number of polycrystalline Cu powder is calculated to be 4.016.

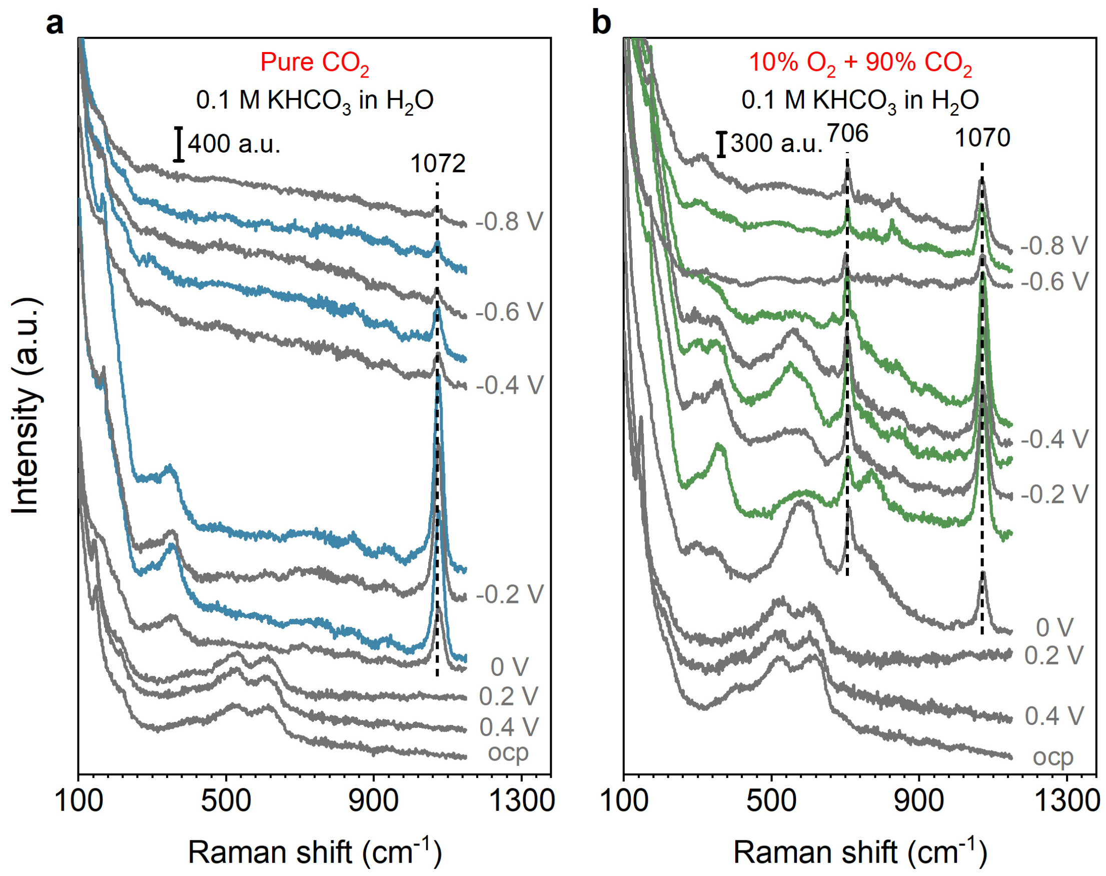

Supplementary Figure 8. Raman spectra of Cu catalyst at electrolysis with (a) pure CO_2 gas feed in 0.1 M $KHCO_3/H_2O$; (b) 10% O_2 + 90% CO_2 gas feed in 0.1 M $KHCO_3/H_2O$.

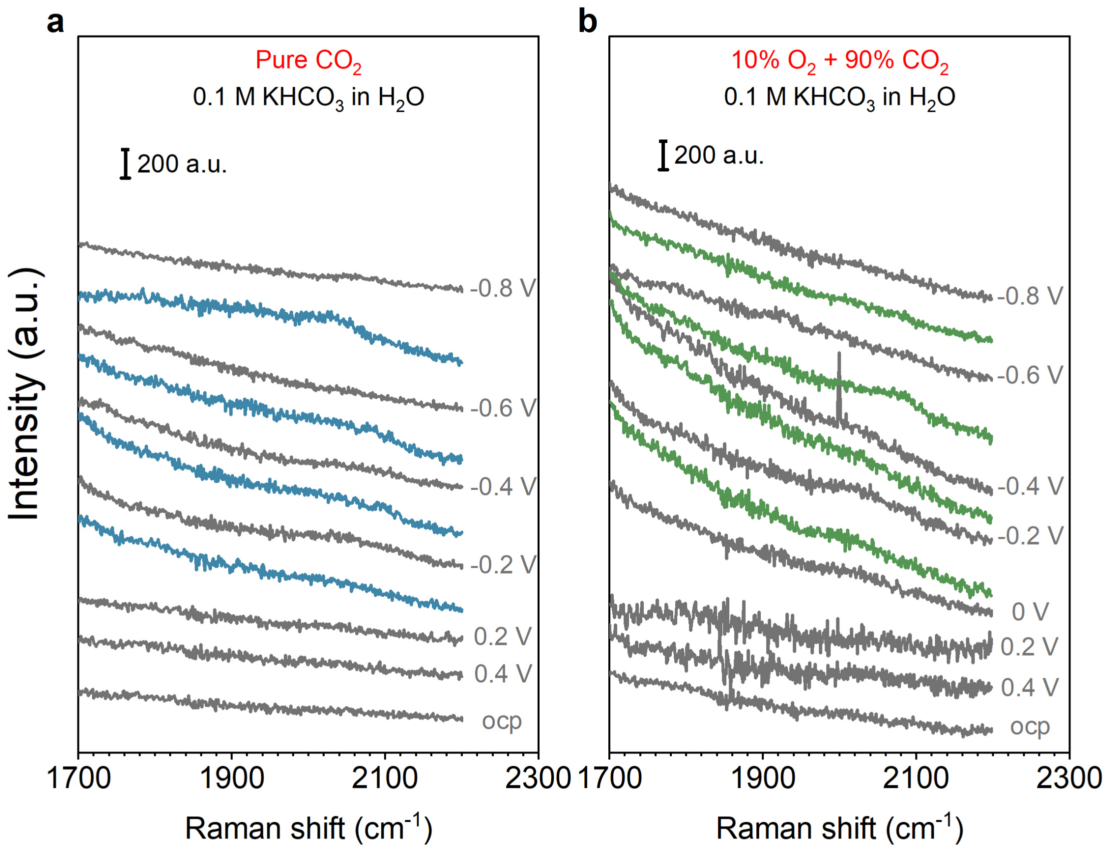

Supplementary Figure 9. The C-O vibration region of Raman spectra of Cu catalyst at electrolysis with (a) pure CO_2 gas feed in 0.1 M $KHCO_3/H_2O$; (b) 10% O_2 + 90% CO_2 gas feed in 0.1 M $KHCO_3/H_2O$.

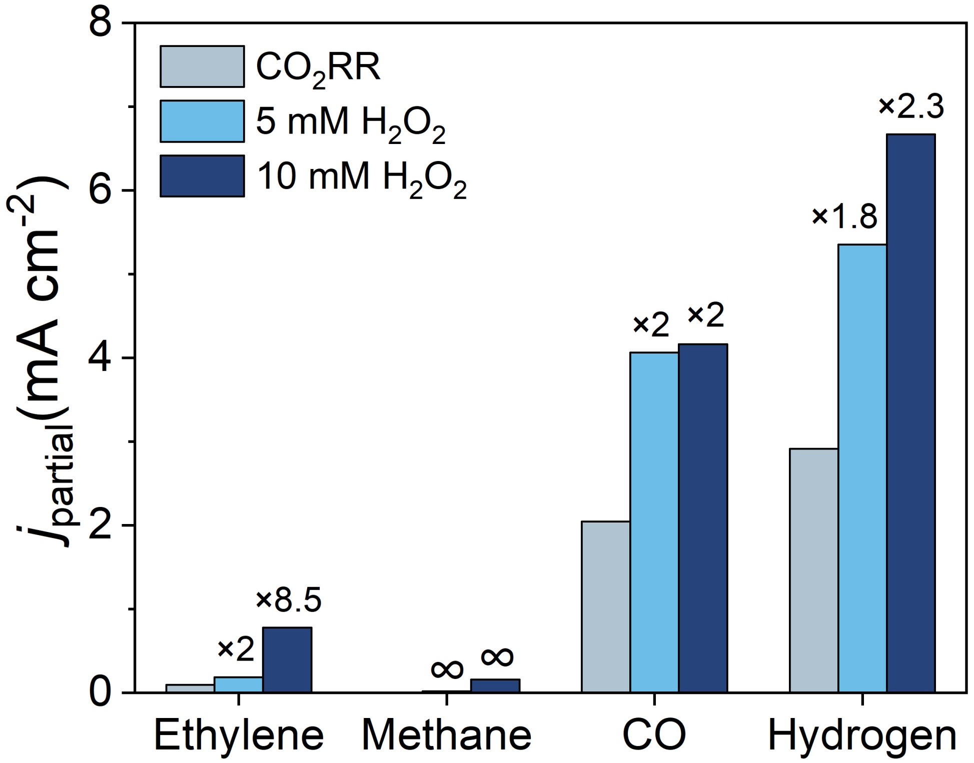

Supplementary Figure 10. Comparison of gaseous products formations on the polycrystalline Cu powder electrode with different H_2O_2 concentrations. The partial current densities are measured at -0.9V$_{RHE}$ in CO_2 saturated 0.1 M $KHCO_3$ solution with 0, 5, 10 mM H_2O_2.

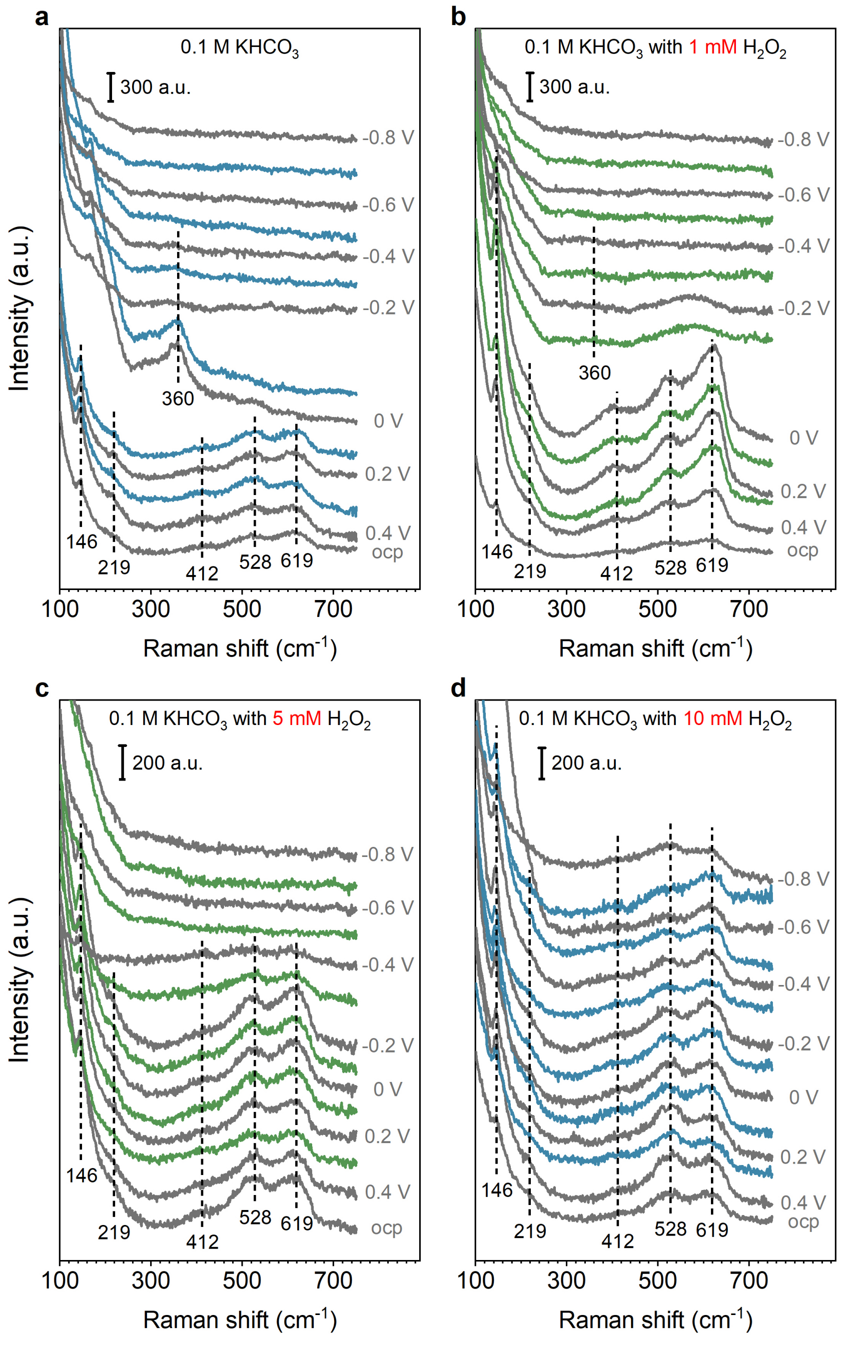

Supplementary Figure 11. In-situ surface-enhanced Raman spectra of polycrystalline Cu powder catalyst at electrolysis in CO_2 saturated 0.1 M $KHCO_3$ solution with (a) 0 mM H_2O_2, (b) 1 mM H_2O_2, (c) 5 mM H_2O_2 and (d) 10 mM H_2O_2.

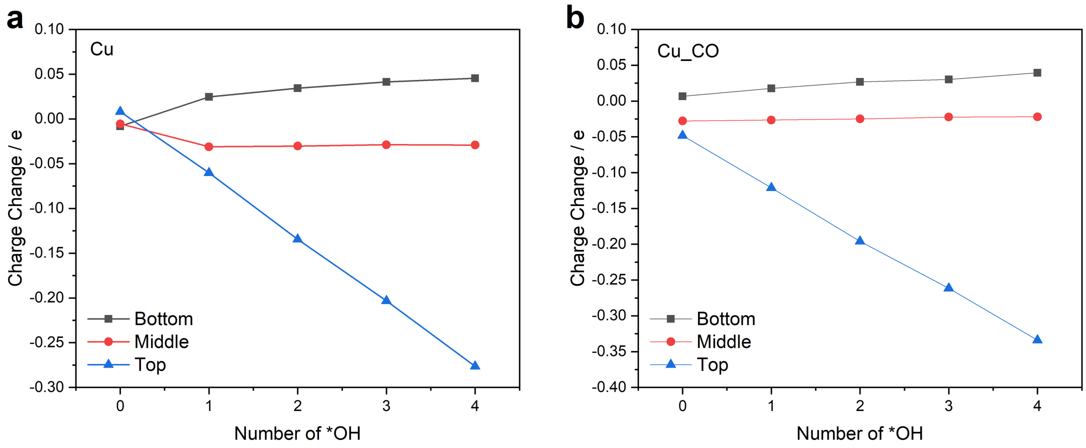

Supplementary Figure 12. Bader charge analysis from DFT calculations for (a) the Cu(100) slab and (b) adsorbed *CO.

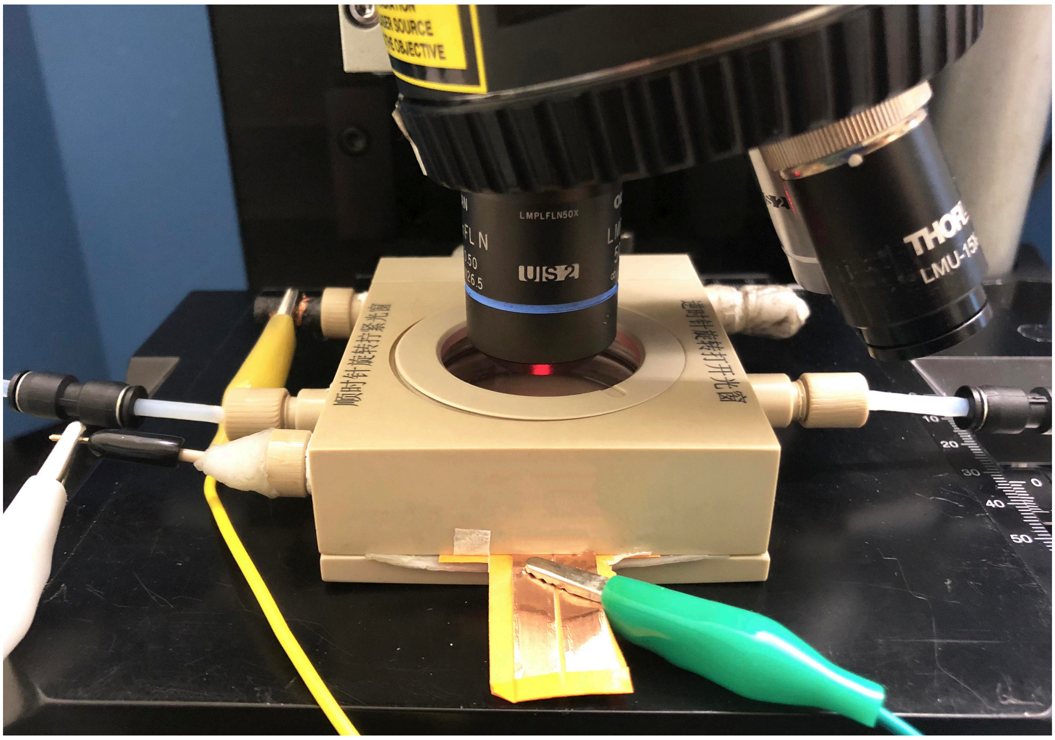

Supplementary Figure 13. Image of the electrochemical cell employed for in-situ surface-enhanced Raman spectroscopy studies.

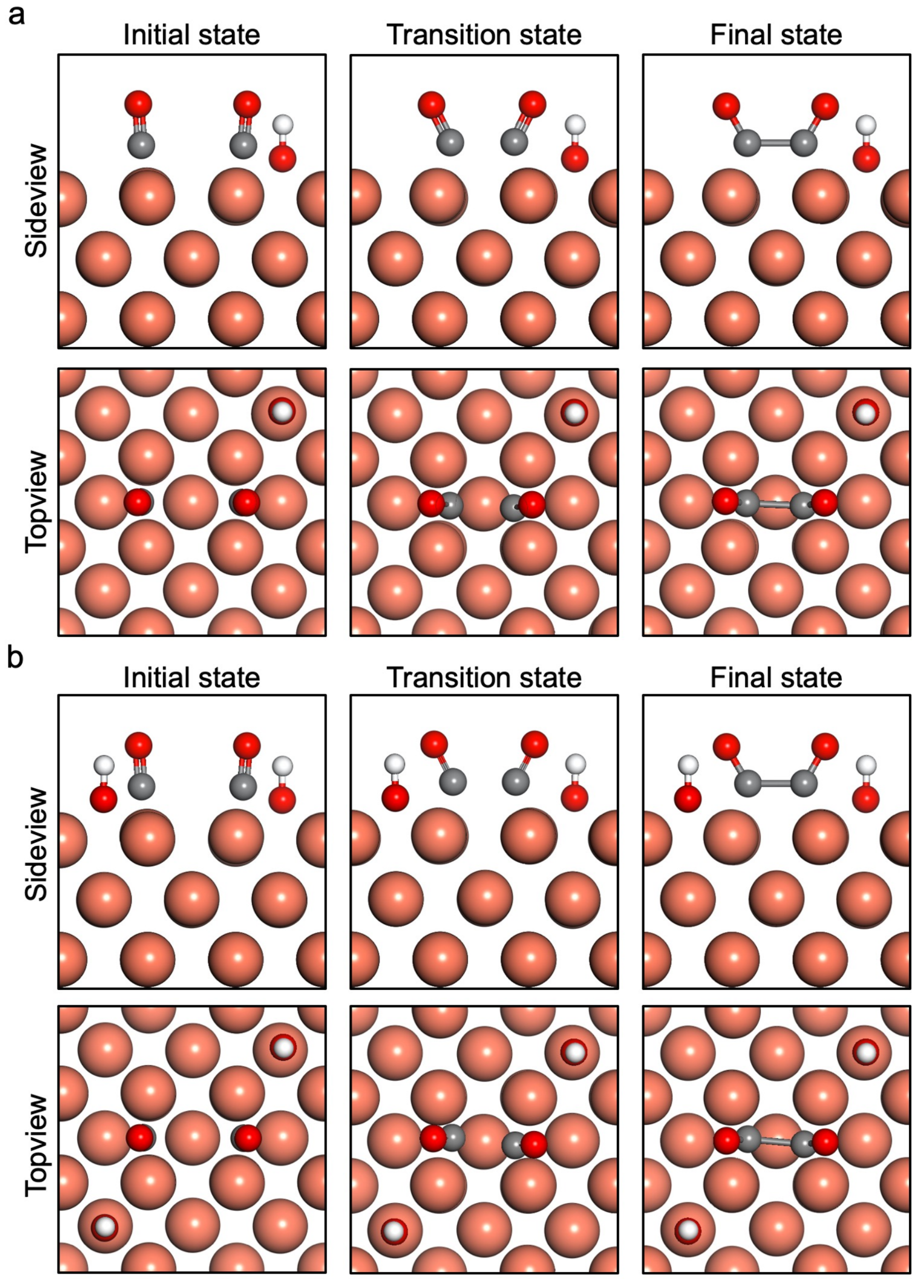

Supplementary Figure 14. Structures of initial states, transition states and final states of *CO dimerization at *OH coverage of (a) 1/9 and (b) 2/9.

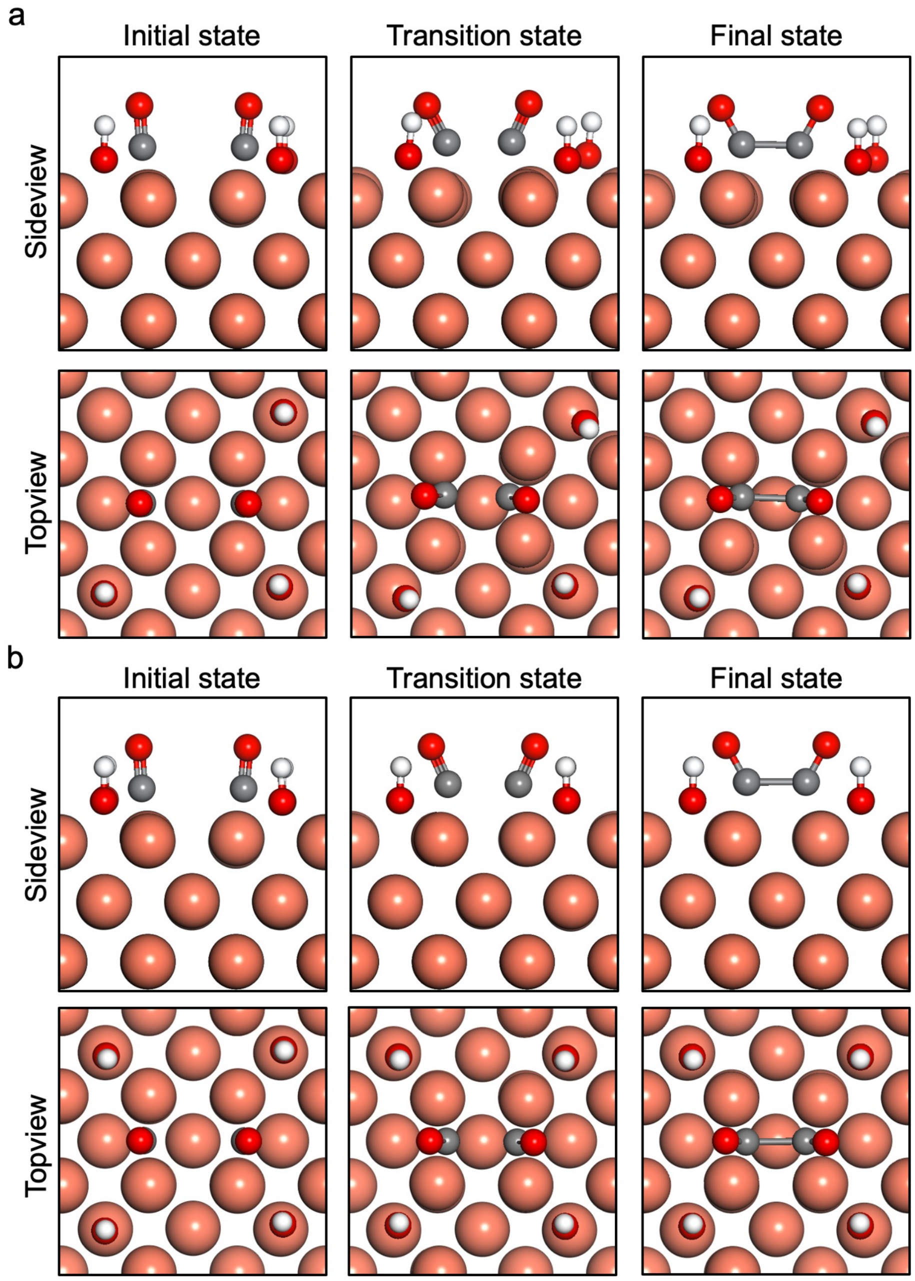

Supplementary Figure 15. Structures of initial states, transition states and final states of *CO dimerization at *OH coverage of (a) 3/9 and (b) 4/9.

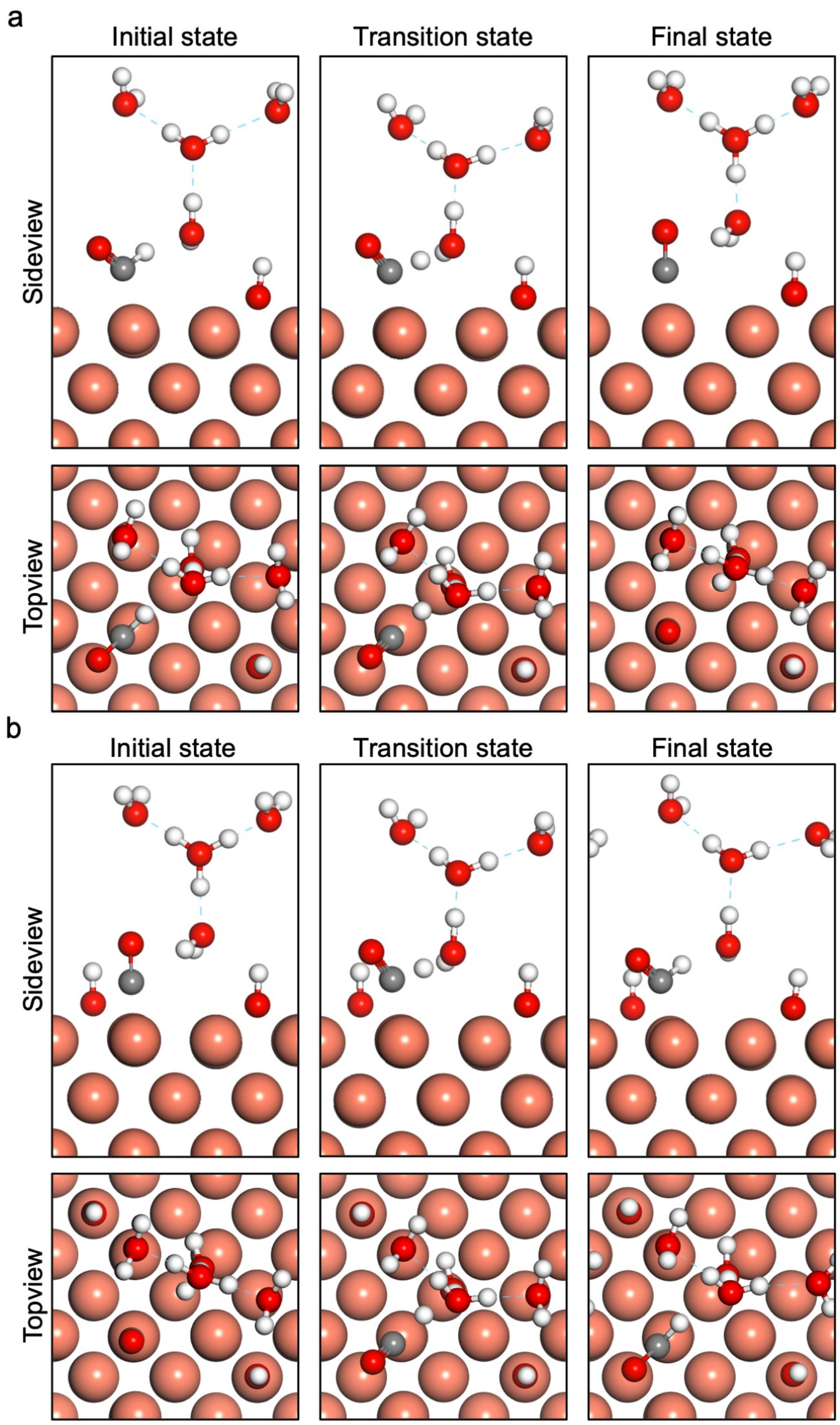

Supplementary Figure 16. Structures of initial states, transition states and final states of *CO hydrogenation at *OH coverage of (a) 1/9 and (b) 2/9.

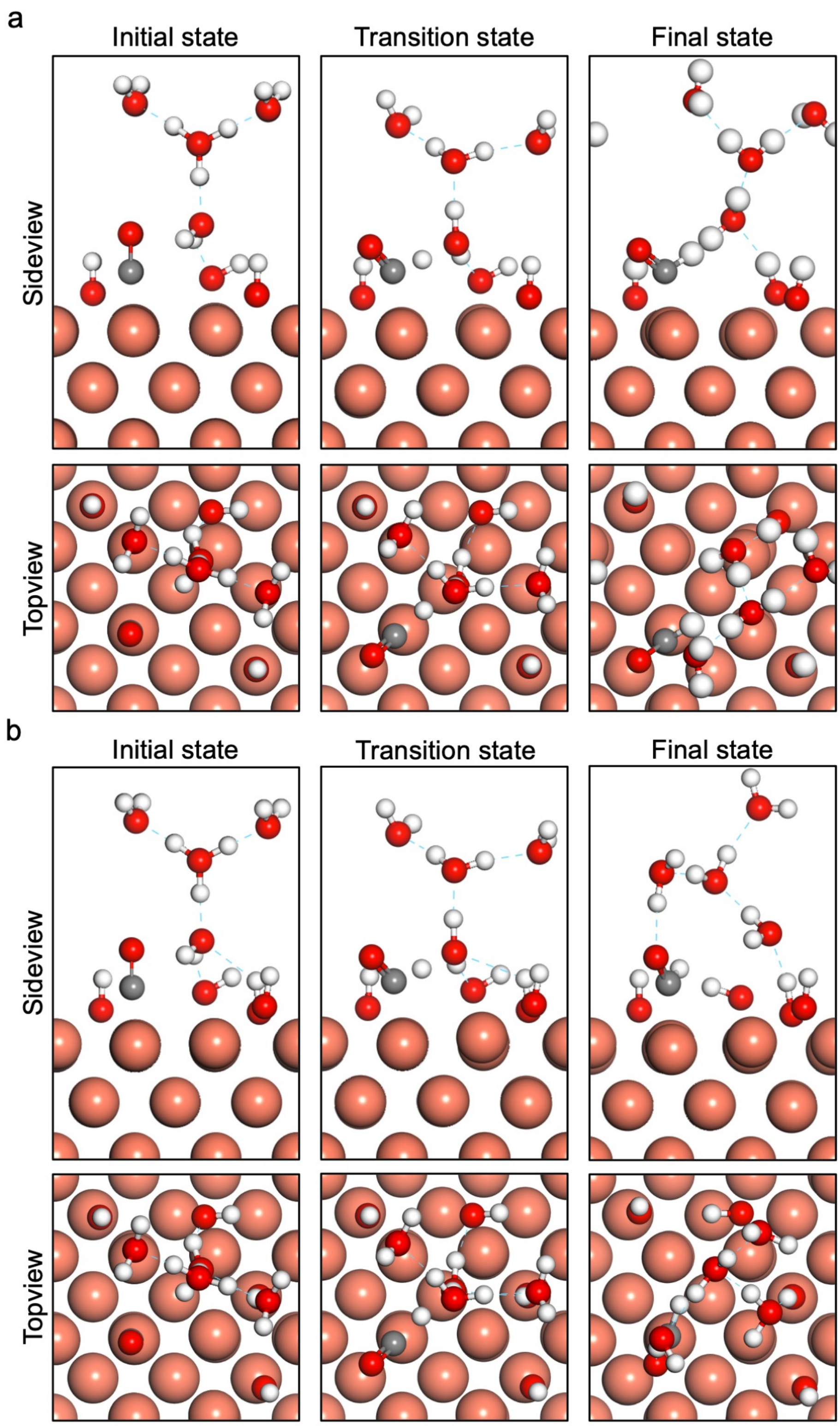

Supplementary Figure 17. Structures of initial states, transition states and final states of *CO hydrogenation at *OH coverage of (a) 3/9 and (b) 4/9.

Supplementary Table 1. The vibrational modes of hollow-site *OH at the initial state of *CO dimerization.

*OH coverage	Mode	Wavenumbers/cm^{-1}
1/9	O-H Bending	656, 623
	Cu-OH Stretching	309
2/9	O-H Bending	684, 658, 627, 592
	Cu-OH Stretching	364, 330
3/9	O-H Bending	699, 671, 648, 647, 603, 589
	Cu-OH Stretching	381, 358, 343
4/9	O-H Bending	730, 703, 681, 677, 648, 635, 614, 570
	Cu-OH Stretching	373, 373, 363, 309

Supplementary Table 2. The vibrational modes of hollow-site *OH at the initial state of *CO hydrogenation.

*OH coverage	Mode	Wavenumbers/cm^{-1}
1/9	O-H Bending	644, 632
	Cu-OH Stretching	305
2/9	O-H Bending	755, 712, 696, 693
	Cu-OH Stretching	329, 310
3/9	O-H Bending	699, 691, 676, 659, 652, 622
	Cu-OH Stretching	343, 336, 328
4/9	O-H Bending	734, 723, 709, 703, 688, 664, 659, 618
	Cu-OH Stretching	381, 351, 340, 334

Supplementary Table 3. The correction terms of free energy.

	Adsorbates	Correction / eV	Adsorbates	Correction / eV	Adsorbates	Correction / eV
*CO dimerization	*CO	0.10	*TS	0.22	*FS	0.24
	*CO+*OH	0.42	*TS+*OH	0.54	*FS+*OH	0.50
	*CO+2*OH	0.73	*TS+2*OH	0.86	*FS+2*OH	0.87
	*CO+3*OH	1.05	*TS+3*OH	1.21	*FS+3*OH	1.22
	*CO+4*OH	1.32	*TS+4*OH	1.51	*FS+4*OH	1.51
*CO hydrogenation	*IS	2.27	*TS	2.47	*FS	2.50
	*IS+*OH	2.66	*TS+*OH	2.81	*FS+*OH	2.94
	*IS+2*OH	2.96	*TS+2*OH	3.14	*FS+2*OH	3.15
	*IS+3*OH	3.23	*TS+3*OH	3.48	*FS+3*OH	3.50
	*IS+4*OH	3.67	*TS+4*OH	3.83	*FS+4*OH	4.00
*OH only	*OH	0.32				
	2*OH	0.61				
	3*OH	0.92				
	4*OH	1.19				

Supplementary Table 4. Faradaic efficiency data of the polycrystalline Cu powder electrodes measured at pure CO_2, 90% CO_2 + 10% O_2, 80% CO_2 + 20% O_2. The standard deviation for each measured product is reported from at least three independent measurements.

Pure CO_2	Faradaic Efficiency (%)							
	Hydrogen	CO	Methane	Ethylene	Formate	Ethanol	Acetate	n-Propanol
-0.75 V	50.680	13.327	0	0.513	24.947	0.897	0.632	0
STD DEV	2.662	2.719	0	0.139	1.172	0.090	0.206	0
-0.80 V	44.873	25.453	0	0.526	23.416	0.664	0.530	0
STD DEV	1.097	2.795	0	0.062	3.451	0.125	0.238	0
-0.85 V	37.385	27.031	0	0.661	26.352	1.593	0.535	0
STD DEV	4.342	5.324	0	0.116	4.467	0.406	0.258	0
-0.90 V	38.439	26.934	0	1.211	26.578	1.855	0.849	1.129
STD DEV	0.796	1.277	0	0.147	1.517	0.209	0.157	0.076
-0.95 V	36.018	28.474	1.135	5.141	23.441	2.653	0.819	1.908
STD DEV	1.338	1.893	0.109	0.743	1.547	0.490	0.099	0.275
-1.00 V	30.918	32.107	3.601	6.611	19.272	2.981	0.856	2.491
STD DEV	1.214	0.518	0.714	0.601	0.205	0.014	0.035	0.014
90% CO_2	Faradaic Efficiency (%)							
+ 10% O_2	Hydrogen	CO	Methane	Ethylene	Formate	Ethanol	Acetate	n-Propanol
-0.75 V	3.721	3.832	0.326	0.608	2.478	1.355	0.682	1.292
STD DEV	0.454	0.472	0.080	0.077	0.528	0.094	0.238	0.217
-0.80 V	4.979	3.316	1.155	2.252	2.268	1.376	0.624	1.077
STD DEV	0.431	0.104	0.256	0.365	0.075	0.062	0.044	0.229
-0.85 V	6.565	3.829	1.430	3.663	3.720	1.912	0.904	1.599
STD DEV	0.745	0.113	0.346	0.421	0.128	0.165	0.149	0.392
-0.90 V	10.826	2.315	5.351	5.738	2.461	2.019	0.819	1.141
STD DEV	0.917	0.219	0.814	0.736	0.216	0.158	0.081	0.108
-0.95 V	11.153	2.641	7.353	6.158	2.395	2.676	1.051	1.255
STD DEV	1.160	0.906	0.260	1.563	0.346	0.147	0.186	0.510
-1.00 V	13.495	2.428	9.460	6.208	2.293	2.482	1.270	1.242
STD DEV	0.535	0.200	0.167	0.742	0.423	0.255	0.059	0.160
80% CO_2	Faradaic Efficiency (%)							
+ 20% O_2	Hydrogen	CO	Methane	Ethylene	Formate	Ethanol	Acetate	n-Propanol
-0.75 V	4.862	2.162	2.313	2.390	2.566	1.458	0.624	0.657
STD DEV	0.317	0.476	0.434	0.117	0.314	0.183	0.278	0.171
-0.80 V	5.402	1.473	4.787	2.689	1.145	1.675	0.948	0.543
STD DEV	0.190	0.180	0.540	0.494	0.326	0.201	0.121	0.039
-0.85 V	8.278	1.510	5.307	4.302	1.653	1.760	1.005	0.916
STD DEV	0.565	0.406	0.448	0.292	0.204	0.074	0.257	0.140
-0.90 V	10.688	1.420	5.421	4.703	0.901	1.773	0.881	1.063
STD DEV	1.066	0.621	0.693	0.539	0.099	0.296	0.075	0.155
-0.95 V	13.163	1.690	5.274	8.040	2.342	2.822	1.089	1.443

STD DEV	1.994	0.544	0.268	0.098	0.529	0.297	0.307	0.384
-1.00 V	18.427	1.663	8.213	6.191	2.071	2.385	0.011	0.862
STD DEV	1.623	0.479	0.988	0.271	0.153	0.274	0.001	0.167

Supplementary Table 5. Partial current density data of the polycrystalline Cu powder electrodes measured at pure CO_2, 90% CO_2 + 10% O_2, 80% CO_2 + 20% O_2. The standard deviation for each measured product is reported from at least three independent measurements.

Pure CO_2	Partial Current Density (mA cm^{-1})							
	Hydrogen	CO	Methane	Ethylene	Formate	Ethanol	Acetate	n-Propanol
-0.75 V	0.933	0.245	0	0.009	0.458	0.017	0.012	0
STD DEV	0.079	0.046	0	0.002	0.003	0.002	0.004	0
-0.80 V	0.997	0.568	0	0.012	0.519	0.015	0.012	0
STD DEV	0.030	0.087	0	0.002	0.055	0.003	0.006	0
-0.85 V	1.699	1.220	0	0.030	1.194	0.072	0.024	0
STD DEV	0.261	0.201	0	0.004	0.199	0.016	0.011	0
-0.90 V	2.915	2.044	0	0.092	2.014	0.141	0.064	0.086
STD DEV	0.110	0.154	0	0.009	0.083	0.020	0.010	0.008
-0.95 V	4.226	3.340	0.133	0.602	2.747	0.311	0.096	0.224
STD DEV	0.263	0.254	0.010	0.079	0.147	0.058	0.010	0.037
-1.00 V	5.096	5.291	0.592	1.088	3.176	0.491	0.141	0.410
STD DEV	0.333	0.224	0.102	0.071	0.117	0.015	0.002	0.009
90% CO_2	Partial Current Density (mA cm^{-1})							
+ 10% O_2	Hydrogen	CO	Methane	Ethylene	Formate	Ethanol	Acetate	n-Propanol
-0.75 V	1.573	1.626	0.137	0.258	1.046	0.575	0.290	0.547
STD DEV	0.125	0.204	0.025	0.032	0.184	0.055	0.099	0.082
-0.80 V	2.636	1.756	0.614	1.195	1.199	0.729	0.331	0.571
STD DEV	0.289	0.137	0.161	0.234	0.050	0.064	0.034	0.132
-0.85 V	3.748	2.183	0.818	2.086	2.120	1.091	0.515	0.913
STD DEV	0.524	0.137	0.217	0.237	0.111	0.125	0.088	0.240
-0.90 V	6.473	1.384	3.202	3.428	1.472	1.208	0.490	0.683
STD DEV	0.434	0.106	0.483	0.371	0.114	0.081	0.040	0.069
-0.95 V	7.061	1.681	4.651	3.920	1.521	1.697	0.669	0.801
STD DEV	0.789	0.604	0.049	1.096	0.264	0.163	0.148	0.138
-1.00 V	9.038	1.624	6.337	4.145	1.528	0.829	1.658	0.850
STD DEV	0.487	0.112	0.295	0.348	0.207	0.068	0.111	0.019
80% CO_2	Partial Current Density (mA cm^{-1})							
+ 20% O_2	Hydrogen	CO	Methane	Ethylene	Formate	Ethanol	Acetate	n-Propanol
-0.75 V	1.573	1.626	0.137	0.258	1.046	0.575	0.290	0.547
STD DEV	0.125	0.204	0.025	0.032	0.184	0.055	0.099	0.082
-0.80 V	2.636	1.756	0.614	1.195	1.199	0.729	0.331	0.571
STD DEV	0.289	0.137	0.161	0.234	0.050	0.064	0.034	0.132
-0.85 V	3.748	2.183	0.818	2.086	2.120	1.091	0.515	0.913
STD DEV	0.524	0.137	0.217	0.237	0.111	0.125	0.088	0.240
-0.90 V	6.473	1.384	3.202	3.428	1.472	1.208	0.490	0.683
STD DEV	0.434	0.106	0.483	0.371	0.114	0.081	0.040	0.069
-0.95 V	7.061	1.681	4.651	3.920	1.521	1.697	0.669	0.801

STD DEV	0.789	0.604	0.049	1.096	0.264	0.163	0.148	0.138
-1.00 V	9.038	1.624	6.337	4.145	1.528	0.829	1.658	0.850
STD DEV	0.487	0.112	0.295	0.348	0.207	0.068	0.111	0.019

Supplementary Note 1. The lack of HCO_3^- or CO_3^{2-} features before the reduction of surface Cu_2O_{surf} (i.e., between the OCP to 0.2 V) could be attributed to the insufficient surface enhancement of Raman signal due to the presence of the native oxide layer on Cu (Supplementary Fig. 8). Only the band at 1072 cm^{-1} attributable to CO_3^{2-} is observed at negative potentials in either the CO_2RR (Supplementary Fig. 8a) or the co-electrolysis (10% O_2 + 90% CO_2, Supplementary Fig. 8b) experiment. The presence of CO_3^{2-} peak and the absence of HCO_3^- peak indicate the high local pH near the surface of our Cu electrodes, which greatly shifts the HCO_3^-/CO_3^{2-} equilibrium towards CO_3^{2-}[3]. With the cathodic potential shift in the CO_2 atmosphere, the intensity of the carbonate band initially increases before gradually decreases at potentials more negative than -0.4 V. The low intensity of the CO_3^{2-} band at more negative potentials, e.g., -0.8 V, is likely due to the strong electrostatic repulsion between negative surface charges and electrolyte anions[4]. With co-feeding O_2, the interfacial pH is increased by the hydroxide produced in the ORR, which is evidenced by the much stronger CO_3^{2-} band at potentials below -0.4 V relative to the maximum peak intensity at -0.4 V (Supplementary Fig. 8b). We note that quantitative interpretation of SERS peak intensity could be complicated by many factors, and thus only qualitative comparisons are made with these two sets of data. The appearance of hydroxyl group at 706 cm^{-1} with the stronger carbonate band suggests a potential correlation, however, additional evidence is needed to support the correlation between the interfacial pH and the formation of the surface hydroxyl group. No obvious CO peak is observed in either CO_2 (Supplementary Fig. 9a) or 10% O_2 + 90% CO_2 saturated (Supplementary Fig. 9b) 0.1 M $KHCO_3$ in this work, which is tentatively attributed to the fast reduction of CO on our Cu electrodes.

Supplementary References

1. Dubot P., Jousset D., Pinet V., Pellerin F. & Langeron J. P. Simulation of the LMM auger spectra of copper. *Surf. Interface Anal.* **12**, 99-104 (1988).
2. Platzman I., Brener R., Haick H. & Tannenbaum R. Oxidation of Polycrystalline Copper Thin Films at Ambient Conditions. *J. Phys. Chem. C* **112**, 1101-1108 (2008).
3. Klingan K.*, et al.* Reactivity Determinants in Electrodeposited Cu Foams for Electrochemical CO2 Reduction. *ChemSusChem* **11**, 3449-3459 (2018).
4. Weitzner S. E.*, et al.* Toward Engineering of Solution Microenvironments for the CO2 Reduction Reaction: Unraveling pH and Voltage Effects from a Combined Density-Functional-Continuum Theory. *J. Phys. Chem. Lett.* **11**, 4113-4118 (2020).
